# Synergistic effect of band convergence and carrier transport on enhancing the thermoelectric performance of Ga doped Cu_2_Te at medium temperatures

**DOI:** 10.1038/s41598-019-43911-2

**Published:** 2019-06-03

**Authors:** Sayan Sarkar, Prashant K. Sarswat, Shrikant Saini, Paolo Mele, Michael L. Free

**Affiliations:** 1 Department of Metallurgical Engineering, University of Utah, Salt Lake City, UTAH 84112 USA; 2 Department of Mechanical and Control Engineering, Kyushu Institute of Technology, Kitakyushu, Japan; 3 Shibaura Institute of Technology, SIT Research Laboratories, Toyosu, Koto-Ku, Tokyo, Japan

**Keywords:** Thermoelectrics, Materials science

## Abstract

Recent advances in high performance thermoelectric materials have garnered unprecedented attention owing to their capability of direct transformation of heat energy to useful electricity. Copper Telluride (Cu_2_Te), a member of the chalcogenide family has emerged as a state-of-the-art thermoelectric material with low thermal conductivity and high thermoelectric (TE) performance, however, this material exhibits exceptional transport properties only at very high temperatures. In this study, we have investigated the synergistic effects of Ga doping on the TE performance by first principles calculations along with experimental validations. The DFT (Density Functional Theory) calculations predicted that Ga doping, within considerable limits enhanced the electrical conductivity and Seebeck coefficients in Cu_2_Te. This proof of concept was validated by experimental synthesis of Ga doped Cu_2_Te by simple direct annealing for shorter durations of 48 hours at 1120 ºC  (~1/4^th^) than in previous work and subsequent thermoelectric characterization. The enhanced electrical conductivity, thermopower, and moderate thermal conductivities led to the optimized TE performance in 3 atomic % Ga doping (Cu_1.97_Ga_0.03_Te), exhibiting a *ZT* value of 0.46 at 600 K, almost three times that of pristine Cu_2_Te in this temperature range. This comprehensive study provides the platform for developing new low-cost and energy efficient TE materials with enhanced *ZT* performance in medium temperature applications.

## Introduction

Environmentally sustainable thermoelectric materials have attracted broad appeal in recent times of energy depletion due to their functional applications in converting heat loss to electricity^1^. In recent times, thermoelectric materials hold the potential to mitigate energy crisis by facilitating lower energy, fuel consumption, and less toxic emissions^2,3^. However, there are a lot of challenges in utilization of widespread thermoelectric devices due to the ever-increasing demand for novel, low cost and environment-friendly thermoelectric materials with high figures of merit^4^. The thermoelectric figure of merit, also known as *ZT* acts as a yardstick for evaluating energy conversion potential^5^ and is a dimensional figure given by $\frac{{S}^{2}\sigma T}{\kappa }$, where *S*, *σ*, *T* and κ refer to the Seebeck coefficient, electrical conductivity, absolute temperature and thermal conductivity (comprising of both lattice and carrier thermal conductivities), respectively^6,7^. Higher *S*, σ and lower κ for any given material improve the thermoelectric performance but these parameters cannot be controlled independently^8–10^ and they bear an inverse relation in metals as given by the Wiedemann-Franz law^11^. Hence, the cornerstone of developing good thermoelectric materials is to tune electron and phonon transport properties by incorporation of resonant doping or atomic-scale percolations^12^. Another effective route already reported in the literature is the development of a phonon-glass substructure (ionic) in a covalent substructure^13^. Carrier transport is significantly promoted by the covalent substructure (Ga/Ge, Co/Sb) leading to higher electrical conductivities, whereas the phonon-glass ionic substructure (Ba^2+^, Ba^2+/^La^3+/^Yb^2+^) averts heat loss (lower thermal conductivity) by accommodating metallic species as were found in case of clathrate^14^ (Ba_8_Ga_16_Ge_30_) and skutterudite^15^ (Ba_0.08_La_0.05_Yb_0.04_Co_4_Sb_12_). Thus, appropriate tuning of the phonon transport properties has been the consistent trend of research over the past decade to enhance thermoelectric properties^16,17^.

Owing to the high carrier mobilities and remarkably low thermal conductivities, transition-metal chalcogenides, specifically tellurides like PbTe, Bi_2_Te_3_ and AgSbTe_2_ have been widely appreciated due to their high thermoelectric performances and exceptional *ZT* values^18–20^. In recent times, copper telluride (Cu_2_Te) has emerged as a promising thermoelectric material with a peak value of *ZT* = 0.3 at 900 K^21^. Other thermoelectric materials in this Cu_2_Te class include copper selenide (Cu_2-δ_Se) and copper sulfide (Cu_2-δ_S) and these materials have exhibited great thermoelectric performance due to their low thermal conductivity. High thermoelectric performances have been reported for these materials with the *ZT* values being 1.5 in copper selenide^22,23^ and 1.7 in copper sulfide^24^ respectively. Compared to Cu_2-δ_Se and Cu_2-δ_S, the bonds in Cu_2_Te are expected to be less ionic due to the lower electronegativity of Te in comparison to Se/S, thus facilitating its carrier conductivity and making its thermoelectric performance more appealing. Cu_2_Te crystallizes in a hexagonal (P6/mmm) crystal structure and has strong Cu-Cu, Te-Te interactions^25^. Several attempts have been made to break or loosen the strong Te-Te or Cu-Cu interactions by doping with foreign elements in order to create a CuTe_4_ substructure that would promote electrical conductivity and/or a high Seebeck coefficient as found in the case of TmCuTe_2_^26^. Another deciding factor for TE performance improvement of a material is also dictated by the density of the materials, which is influenced by direct annealing or hot-pressing techniques like SPS (Spark Plasma Sintering). A recent investigation showed that directly annealed samples of Cu_2_Te without SPS significantly optimized electrical transports with a realization of large power factors accompanied by low thermal conductivities^27^. By direct annealing of Cu_2_Te pellets, the enhanced electrical transport and reduced thermal conductivity made it possible to achieve high *ZT* values of 1.1 at 1000 K, which classifies it as a top thermoelectric material^27^.

**Figure 1:**
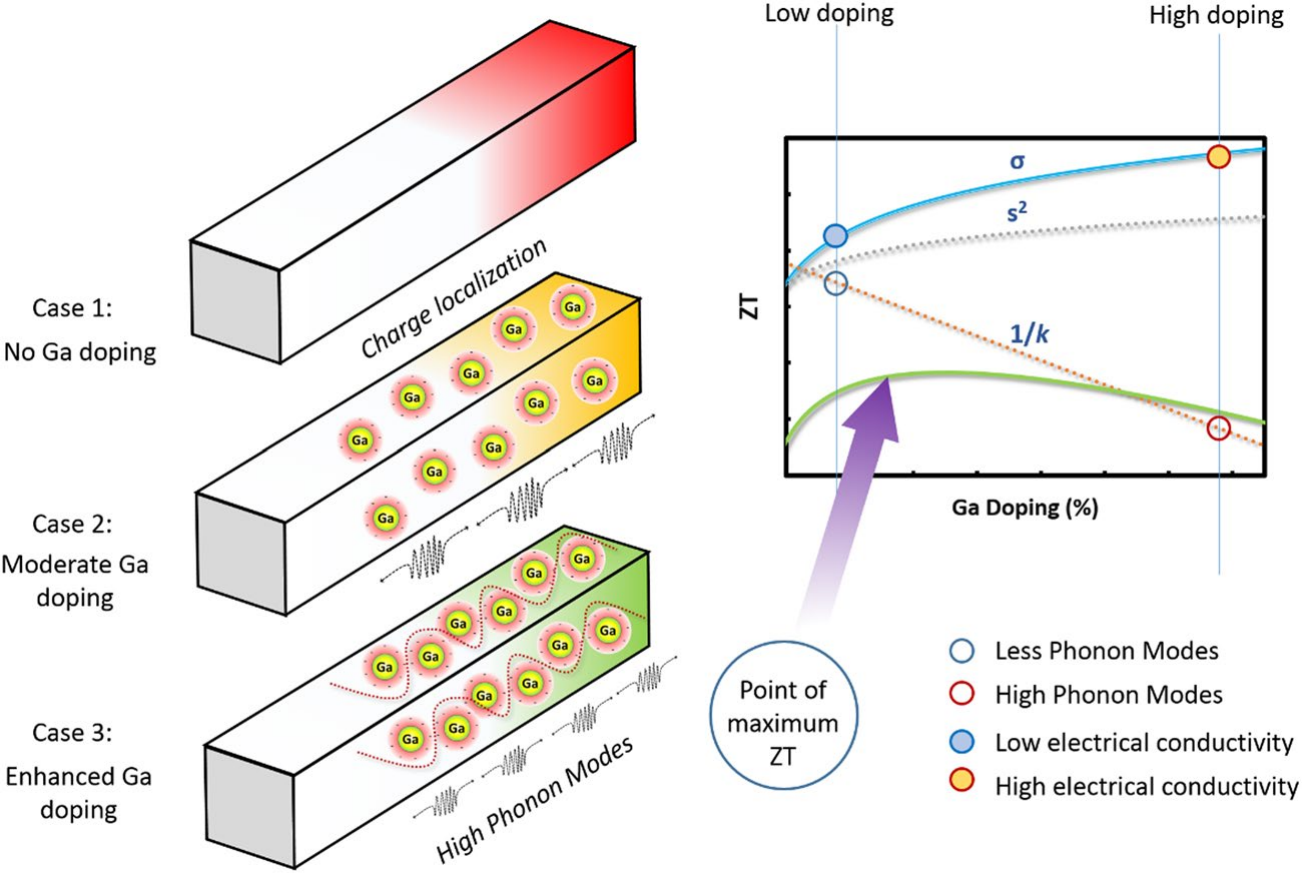
Understanding the effect of Ga addition on thermoelectric performance of pristine and Ga-doped Cu_2_Te. Synergistic effects of Ga doping in Cu_2_Te within certain limits (moderate doping ~3%) resulting in a large conductivity (σ), optimized thermopower (S), moderate thermal conductivity (κ) performance, thereby extensively enhancing the *ZT* of Cu_2_Te at medium temperatures. With the increase of Ga doping content, there was an enlarged carrier conductivity (σ), due to the injection of immense p-type carriers assuming the shape of a “hump” in the valence band minimum (VBM). Subsequently, the minority charge carriers (e−) got coupled with the dopant Ga^3+^ ions forming localized cloud of charge carriers. On the application of a temperature gradient, this charge localization enhanced the thermopower (S) of the device significantly. However, Ga doping also exerted an adverse effect if it is not restricted within certain limits owing to the increased number of thermally active phonon modes in disordered species (CuGaTe_2_) resulting in higher κ (enhanced doping ~5%). Since *ZT* = $\frac{{S}^{2}\sigma T}{\kappa }$, there exists a trade-off between enlarged electrical transport (σ, S) & heat transport (κ) coefficients as a consequence of Ga doping. Optimization of the TE efficiency *ZT* was achieved at moderate Ga doping levels (~3%), as shown by arrow, owing to a substantial σ, large S and moderate κ.

In this study, we adopt a synergistic combination of these two approaches- high temperature annealing and incorporation of a foreign dopant Ga in Cu_2_Te in order to enhance its thermoelectric performance (See Fig. 1). Introduction of Ga^3+^ as a shallow n-type donor leads to the partial substitution of Cu^+^, thus promoting electrical conductivities significantly along with breaking strong Cu-Cu interactions in Cu_2_Te^28^. Doping of Ga, in addition, promotes the generation of lighter bands leading to band convergence and enhanced thermoelectric performance^29^. In heavily doped semiconductors, due to the presence of light bands at the conduction band minimum leads to the accumulation of a large number of electrons in the secondary conduction band, which enhances the Seebeck coefficient and thermoelectric performance^29^. Moreover, the close proximity of ionic and covalent radii of Ga (1.30, 1.26 Å) with Cu (1.35, 1.38 Å) would also foster less lattice distortions and enhanced transport^30,31^. These two synergistic effects of direct annealing and incorporation of Ga as a shallow n-type donor on improving the thermoelectric properties of Cu_2_Te formed the basis of our study. By first-principles density functional theory and thermoelectric transport calculations, the improved electrical conductivity, density of states, Seebeck coefficient and enhanced *ZT* values as a consequence of Ga doping, was predicted. It was followed by synthesis of Ga doped Cu_2_Te pellets by direct annealing and experimental validation of the enhanced thermoelectric properties as a result of doping. This simple, energy-efficient and environment-friendly direct annealing treatment along with Ga doping of Cu_2_Te results in enhanced thermoelectric performance at medium temperatures which may be beneficial to utilize waste heat energy generated in various automotive and manufacturing industries in the range of 400–900 K^32^.

## Results and Discussion

### Density functional theory electronic structure results

**Figure 2:**
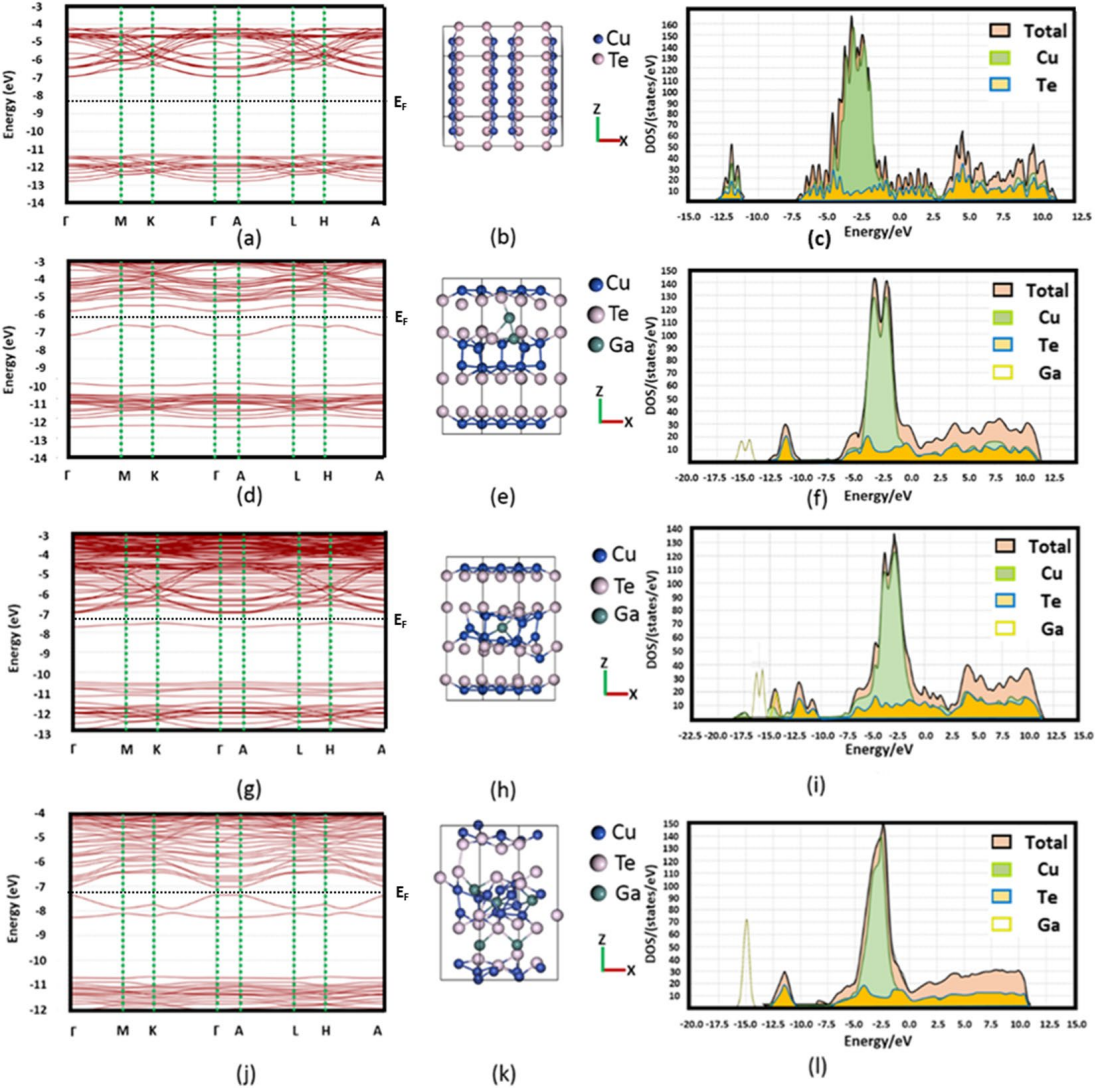
Crystal structure, DFT based band structure and Density of States (DOS) diagram of pristine and Ga-doped Cu_2_Te. Quantum Expresso based bandstructures (with PBE optimization) and without spin orbit coupling (SOC) for (**a**) Cu_2_Te (**d**) Cu_1.99_Ga_0.01_Te (**g**) Cu_1.97_Ga_0.03_Te (**j**) Cu_1.95_Ga_0.05_Te. The bandstructures were simulated along the high symmetry hexagonal directions Γ → M → K → Γ → A → L → H → A. Top view of 3 × 2 × 2 supercell of primitive unit cell of hexagonal lattice (space group P6/mmm) of the crystals of (**b**) Cu_2_Te (**e**) Cu_1.99_Ga_0.01_Te (**h**) Cu_1.97_Ga_0.03_Te (**k**) Cu_1.95_Ga_0.05_Te. Normalized Density of States diagram for (**c**) Cu_2_Te (**f**) Cu_1.99_Ga_0.01_Te (i) Cu_1.97_Ga_0.03_Te (**l**) Cu_1.95_Ga_0.05_Te.

We have presented the analysis of the electronic properties of doped and pristine Cu_2_Te in order to understand the influence of doping of Ga on the transport properties. Table 1 indicates the optimized crystal structure, lattice information and the total energy of the supercells of Cu_2_Te, Cu_1.99_Ga_0.01_Te, Cu_1.97_Ga_0.03_Te, Cu_1.95_Ga_0.05_Te, and Cu_2_Ga_0.01_Te_1.99_. For the better understanding of the site that Ga would displace because of doping, first principles calculations were performed by considering the two stoichiometries Cu_1.99_Ga_0.01_Te and Cu_2_Ga_0.01_Te_1.99_. As indicated in Table 1, Cu_1.99_Ga_0.01_Te was found to be more stable than Cu_2_Ga_0.01_Te_1.99_ with the total energy of the supercell being 4104.73 Ry. This can be attributed to the presence of too many Cu vacancies in Cu_2-x_Te as defects, which have a low activation of energy^27^ and can be substituted by Ga. After structural optimizations, all the crystals structures after doping were found to be the same with respect to the parent Cu_2_Te: hexagonal (space group-P6/mmm) as shown in Fig. 2(b–k) and Table 1. Due to the resemblance of ionic and covalent radii of Ga (1.30, 1.26 Å) with Cu (1.35, 1.38 Å), there was no distortion in the crystal structure with lattice strains along the a, c-axes being restricted to <1% after doping. However, after the incorporation of Ga, changes in the occupation site of Cu and Ga were observed, which affected the band structure by decreasing the effective mass. The substitution of n-type donor Ga^3+^ in the Cu vacancies would foster higher carrier density and hence higher electrical conductivity in the system. At a high Ga doping of 5 atomic %, there was more accumulation of free electrons near the conduction band minimum, as a result, the Fermi level moved upward resulting in band convergence. Due to the doping of Ga, multiple conduction and edges were induced which helped in the reduction of *∆E* (band offset) (please see Fig. 2d,g,j) and facilitated carrier transport by offering additional channels. There was more convergence of conduction band valleys at *Г*, *M*, *L* and *A* points of the Brillouin zone resulting in band degeneracy which enhanced carrier transport and the gauge factor (B) dictating thermoelectric performance^33^. The optical band gap measurements (shown in Fig. S5) also confirmed this band convergence showing that optical band gap decreased as Ga content increased indicating the enhancement of carrier concentration.

**Table 1:** The stoichiometric formula, crystal structure, lattice information and total energy of the supercells of the following compounds

Compound	CRYSTAL STRUCTURE	SPACE-GROUP	LATTICE PARAMETERS						Total energy
			a (Å)	b(Å)	c (Å)	α	β	γ	Ry
Cu_2_Te	Hexagonal	*P6/mmm* (No. 191)	8.608	8.608	17.05	90°	90°	120°	−4148.55
Cu_1.99_Ga_0.01_Te	Hexagonal	*P6/mmm* (No. 191)	8.613	8.613	17.03	90°	90°	120°	−4104.73
Cu_1.97_Ga_0.03_Te	Hexagonal	*P6/mmm* (No. 191)	8.618	8.618	17.08	90°	90°	120°	−4260.91
Cu_1.95_Ga_0.05_Te	Hexagonal	*P6/mmm* (No. 191)	8.623	8.623	17.11	90°	90°	120°	−4430.52
Cu_2_Ga_0.01_Te_1.99_	Hexagonal	*P6/mmm* (No. 191)	8.611	8.611	17.01	90°	90°	120°	−4103.81

The band structure and electronic DOS (Density of States) was calculated for all the crystals, considering their entire hexagonal Brillouin zone (shown in Fig. 5l), which encompassed all of the following points Γ(0, 0, 0), A (0, 0, π/c), K(4π/3*a*, 0, 0), H (4π/3*a*, 0, π/c), M(π/*a*, −π/√3*a*, 0) and L(π/*a*, −π/√3*a*, π/c). Figure 2 shows the calculated band structures (without spin-orbit coupling) and DOS for Cu_2_Te, Cu_1.99_Ga_0.01_Te, Cu_1.97_Ga_0.03_Te and Cu_1.95_Ga_0.05_Te. The band structures revealed that there was a significant reduction in the bandgap as Ga content increased in the crystal lattice (please refer to Fig. 2a,d,g,j). With the increase of Ga content to 1% and 3% (Fig. 2d,g), there was an accumulation of free electrons near the conduction band minimum at the center of the crystal (Γ) resulting in the formation of n-type narrow band semiconductors. Moreover, there was an observation of band inversion in the vicinity of the Fermi-level with the increase of Ga content, along with the Fermi energy level (*E*_*F*_) overlapping with the partially filled bands. As the Ga doping increased to 5%, it may be interesting to note, the band energy dispersion followed a Dirac-cone (please refer Fig. 2j). This may be attributed to the topologically robust states present in the hexagonal Newtony structures^34,35^, which is similar to hexagonal Cu_2_Te. Another intriguing feature, which is an effect of doping of Ga was the emergence of steeper bands near the Fermi-energy, which subsequently reduced the effective mass of the bands^36^ (especially in the case of 5% Ga doping). The lighter bands promoted better electrical conductivity, since the effective band mass bears an inverse relation with the conductivity (σ) of an isotropic parabolic band (${\rm{\sigma }}=\frac{n{e}^{2}\Gamma }{{m}^{\ast }}\,$, *n* = carrier concentration, m* = *m*_B_ is the effective mass and г(*E*) = *E*^r^ is the scattering time, and *r* is the scattering parameter.)

The electronic DOS of the doped and pristine Cu_2_Te (please refer to Fig. 2c,f,i,l) were calculated to understand the re-distribution of charge carriers because of doping. The partial DOS for Cu_2_Te indicated that the conduction band was comprised of Cu 3d and Te 5p orbitals (Fig. 2c). Doping of 1% Ga as a shallow p-type acceptor resulted in a small “hump” in the electronic DOS valence band (Fig. 2f), which was mainly composed of Ga 4 s and 4p orbitals^28,37^. As the doping of Ga increased to 3% and 5%, further addition of electrons near the CBM augmented the hump composed 4 s and 4p orbitals (please see Fig. 2i,l). It was also revealed in the case of Cu_1.95_Ga_0.05_Te, the elecctron pockets assumed a corrugated shape around the Γ point, indicating an increase in the Fermi surface area which promoted higher electrical conductivity^37^. Due to band convergence of conduction band minima and valence band maxima, these bands contributed more substantially to thermoelectric transport and improvement of *ZT*^38^.

### Density functional theory transport calculations

Calculation of the thermoelectric coefficients was conducted based on the band dispersions using the opensource *LanTrap* tool that provides a solution to the Boltzmann Transport Equation (BTE) assuming a relaxation time approximation. The transport coefficients were calculated based on the following approach:

1 $$\sigma ={\int }_{-\propto }^{+\propto }{\sigma }\,({E}){dE}$$

2 $$S=-\,\frac{1}{{qT}}\,\frac{{\int }_{-\propto }^{+\propto }({E}-{{E}}_{{F}}){\sigma }\,({E}){dE}}{{\int }_{-\propto }^{+\propto }{\sigma }\,({E}){dE}}\,$$

3 $${{\kappa }}_{0}=\frac{1}{{{q}}^{2}{T}}{\int }_{-\propto }^{+\propto }{({E}-{{E}}_{{F}})}^{2}{\sigma }\,({E}){dE}$$

4 $${{\kappa }}_{{e}}={{\kappa }}_{0}-T{\sigma }{{S}}^{2}$$

5 $${\sigma }\,({E})={{q}}^{2}{\Xi }({E})\,(\,-\,\frac{\partial {{f}}_{0}}{\partial {E}})$$

where, σ = electronic conductivity, *S* = Seebeck coefficient, *κ*_*e*_ = electronic thermal conductivity, σ (E) = differential electronic conductivity, *E* = band dispersion energy, *E*_*F*_ = Fermi energy, *q* = electronic charge, *T* = absolute temperature, f_0_ = function related to the transport coefficients. In equation (5), the transport function part of the BTE equation, Ξ(E) is related to the average number of thermally active phonon modes per cross-sectional area as

6 $${\Xi }({E})=\frac{2}{{h}}\frac{{M}({E}){\lambda }({E})}{{A}}$$

where, *h* = Planck’s constant, *A* = cross-sectional area, *λ*(*E*) = mean free path for scattering. The average number of thermally active phonon modes was again calculated based on the DFT computed band structure using *LanTrap* at a definite temperature as

7 $$\langle {M}\rangle ={\int }_{-\propto }^{+\propto }{M}({E}){W}({E}){dE}$$

*W*(*E*) is the normalized “window function” following Bose-Einstein distribution^39^. The thermal conductivity can also be related to the average number of thermally active phonon modes and the phonon mean free path for scattering by this formula^39^

8 $${\kappa }={{\kappa }}_{0}{\int }_{-\propto }^{+\propto }{M}({E}){W}({E},{T}){\lambda }({E},{T}){dE}$$

κ_0_ being a constant also known as a quantum of thermal conductance.

Based on equation (8), it can be understood that thermal conductivity is heavily dependent on the average number of thermally active phonon modes *M* among all the other transport coefficients. The other factor which directly influences the thermal conductivity is the mean free path for phonon (MFP) scattering and is related to the band structure dispersion and scattering mechanism. This MFP for any crystal was found to be heavily dependent on the anisotropy and the layer thickness^39^, in our study we have assumed isotropic, similar supercells in our band structure calculations thus neglecting the contribution from MFP on thermal conductivity.

**Figure 3:**
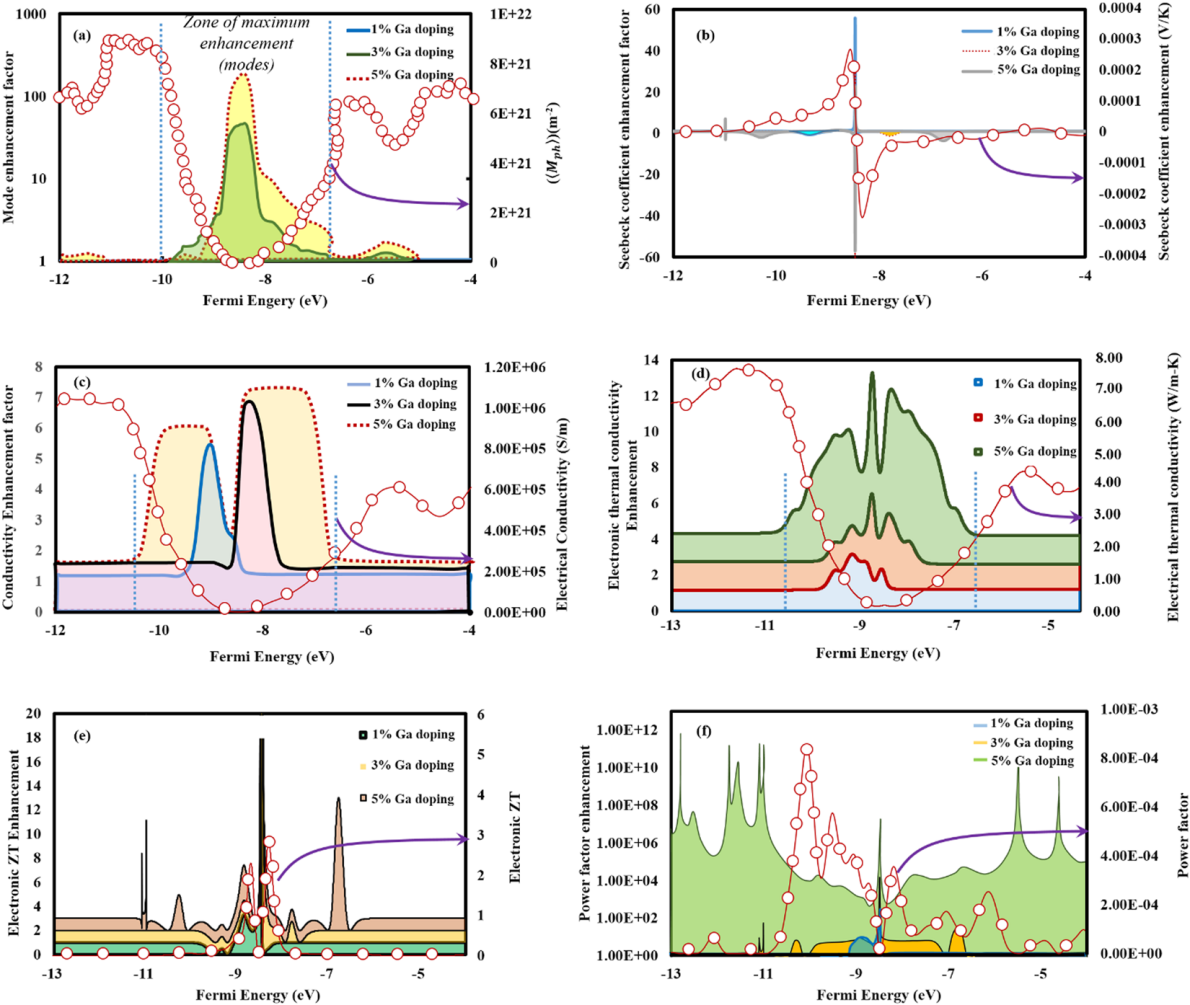
First principles calculations of enhancement factor in distribution of thermally active phonon modes and transport coefficients after doping from the band dispersions of pristine and Ga-doped Cu_2_Te. Enhancement factor (shown by colored area plots) with respect to pristine Cu_2_Te as a function of the Fermi energy in the (**a**) average number of conducting phonon modes per cross-sectional energy (*M*_*ph*_) (**b**) Seebeck coefficient (S) (**c**) Electronic conductivity (σ) (**d**) Electronic thermal conductivity (κ) (**e**) electronic *ZT* (**f**) power factor (PF) after doping of 1, 3 and 5 atomic % Ga (shown in the legends). The magnitude of *M*_*ph*_, S, σ, κ, *ZT* and PF for pristine Cu_2_Te has been plotted (line plots with round markers) with their magnitudes represented in the secondary Y-axis for comparison. The absence of colored area regions for a particular plot (doping) essentially represents zero enhancement in that zone of Fermi energy. These distributions of modes and transport coefficients were calculated from the bandstructures of the corresponding crystals using the thermoelectric transport calculator tool *LanTrap*.

Figure 3 shows the enhancement in transport property coefficients with respect to pristine Cu_2_Te as a function of the Fermi energy, whereas Figs S1 and S2 represent their actual values calculated from first principles calculations. Figure S1 shows the *M* for pristine and doped Cu_2_Te for the band energy dispersion and Fig. 3(a) shows the enhancement in *M* as a function of the Fermi energy after doping of 1%, 3% and 5% Ga. The distribution of modes followed an inverted pattern for Cu_2_Te with significantly less distribution in the energy range of the band gap (−6 to −11 eV) as shown in Fig. 3a (line plot with round markers). As doping of Ga is incorporated, the population of thermally active phonon modes increased considerably in the energy range of the VBM (−11 to −9.5 eV) for 1% and 3% doping (Figs 3a and S1b,c). Doping of 5% Ga (See Fig. S1d) further increased the *M* in the energy range of the CBM and the VBM, consistent with the pattern that Ga doping introduced more electrons near the VBM (*M*) resulting in the tapering of the band gap. Enhancement in the thermally active phonon modes was most prominent in case of 3% and 5% doping of Ga between the energy zones of VBM and CBM due to the introduction of electrons (Fig. 3a). Since *M* is directly related to the thermal conductivity, the increased number of thermally active phonon modes in Ga doped Cu_2_Te would promote heat dissipation, thus degrading the thermoelectric performance. It is thus interesting to note that although Ga doping promoted the transport coefficients like electrical conductivity and Seebeck coefficient, it manifested a counter effect on the electronic thermal conductivity reducing thermoelectric conversion efficiency.

We computed the electronic transport coefficients Seebeck coefficient (*S*), electronic conductivity (σ), electronic thermal conductivity (*κ*), power factor (*S*^2^*σ*) and the electronic *ZT* ($\frac{{S}^{2}\sigma T}{\kappa }$) for pristine and doped Cu_2_Te at 300 K from the distribution of modes. Figure S2(a) shows the electronic conductivities of Cu_2_Te, Cu_1.99_Ga_0.01_Te, Cu_1.97_Ga_0.03_Te and Cu_1.95_Ga_0.05_Te. It was observed that the electronic conductivities increased significantly with the increase in doping content of Ga (Fig. 3c). There were a lot of intrinsic vacancies in Cu_2-x_Te^27^, which were substituted by the Ga^3+^ n-type donor contributed three electrons to the system leading to the initiation of the large number of free electrons, particularly near the conduction band maxima (CBM). The peak electronic conductivity of 2.75 × 10^6^ S/m was achieved in case of Cu_1.95_Ga_0.05_Te in the vicinity of VBM, the magnitudes of *σ* decreased in the CB compared to the valence band and exhibited the highest depletion near the Fermi level due to the absence of charge carriers. The highest enhancement factor in electronic conductivity was around 7 for Cu_1.95_Ga_0.05_Te, as shown in Fig. 3c. The same trend of variation of electronic conductivity was also observed in the case of, Cu_1.99_Ga_0.01_Te, and Cu_1.97_Ga_0.03_Te with conductivities increasing with the Ga content. The Seebeck coefficients, however, did not exhibit an increasing trend with increasing electron concentration as a result of doping (Fig. S2b). The composition Cu_1.99_Ga_0.01_Te showed the maximum Seebeck coefficient value of *S* = 365 μV/K in the energy range −9 to −8 eV (please refer to Fig. S2b), much higher than the peak *S* value of pristine Cu_2_Te at 745 K (50 μV/K)^25^. Maximum values of positive Seebeck coefficients were obtained in the same energy −9 to −8 eV for all the other compositions, with Cu_1.95_Ga_0.05_Te exhibiting the second highest *S*_peak_ value of 310 μV/K, followed by Cu_2_Te (*S*_peak_ = 255 μV/K) and Cu_1.97_Ga_0.03_Te (*S*_peak_ = 240 μV/K). Compared to pristine Cu_2_Te, the highest enhancement factor of ~58 in Seebeck coefficient was observed in Cu_1.99_Ga_0.01_Te followed by 40 times enhancement in Cu_1.97_Ga_0.03_Te (Fig. 3b).Thus the enhancement in Seebeck coefficients did not occur as a function of increasing Ga concentration unlike the electronic conductivity, such behavior may be attributed due to the trade-off between S and σ to maximize the power factor^40^.

The electronic thermal conductivities (*κ*) were then plotted as a function of Fermi energy at 300 K, which exhibited similar trends with electronic conductivities (*σ*) (Fig. S2c). In all the compositions, the κ values were significantly greater in the VB than CB, the least values of the same were achieved in the vicinity of the Fermi energy range of −8 to −9.5 eV (see Fig. S2c). Pristine Cu_2_Te exhibited an average thermal conductivity 3.7–4 W m^−1^ K^−1^, consistent with the experimental κ_total_ values of Cu_2_Te^25^ (4.58–2.77 W m^−1^ K^−1^). The κ values increased with the increasing Ga content with the peak value of 15 W m^−1^ K^−1^ being achieved in Cu_1.95_Ga_0.05_Te at the Fermi energy ~−10 eV. The zone between the VBM and CBM showed the highest increase in electronic thermal conductivities represent by colored plots for all compositions, the maximum enhancement factor reached up to 13 in Cu_1.95_Ga_0.05_Te (Fig. 3c). Such an increase in electronic thermal conductivities with increasing Ga content was consistent with the increased number of thermally active phonon modes *M*, which bears a direct relation with electronic thermal conductivity (equation 8). This behavior could be attributed to the formation of disordered ionic species after Ga doping, which facilitated easy dissipation of heat, thus increasing the electronic thermal conductivities. After calculation of *S*, σ & κ, we computed the thermoelectric figures of merit, *ZT* for all the four compositions. As shown in Fig. S2d, electronic *ZT* values were found to be much higher in the energy range of −9 to −8.5 eV (near VBM) than in the energy range of −8.5 to −8 eV (near CBM). The peak *ZT* of 5.7 was achieved in the case of Cu_1.97_Ga_0.03_Te due to its least thermal conductivity followed by Cu_1.99_Ga_0.01_Te, Cu_1.95_Ga_0.05_Te, and Cu_2_Te. With respect to Cu_2_Te, maximum enhancement in *ZT* values of up to 18 was observed in Cu_1.97_Ga_0.03_Te, followed by the other compositions Cu_1.99_Ga_0.01_Te, and Cu_1.99_Ga_0.01_Te 9 (Fig. 3e). It must be remembered that these *ZT* values calculated from first principles were significantly higher than the experimental values of *ZT* of Cu_2_Te^27^ (0.55–1.1), since these transport calculations neglected optical phonon scattering and lattice vibrations, thereby underestimating the relaxation time for scattering^40^. But these calculations successfully assisted us in predicting that optimum *ZT* value was obtained at 3% Ga doping, after which the *ZT* values showed a decreasing trend due to the formation of disordered species which would easily liberate owing to the enhanced vibrations of phonon modes. This dissipation of heat would then subsequently increase the thermal conductivity and adversely affect the thermoelectric performance.

**Figure 4:**
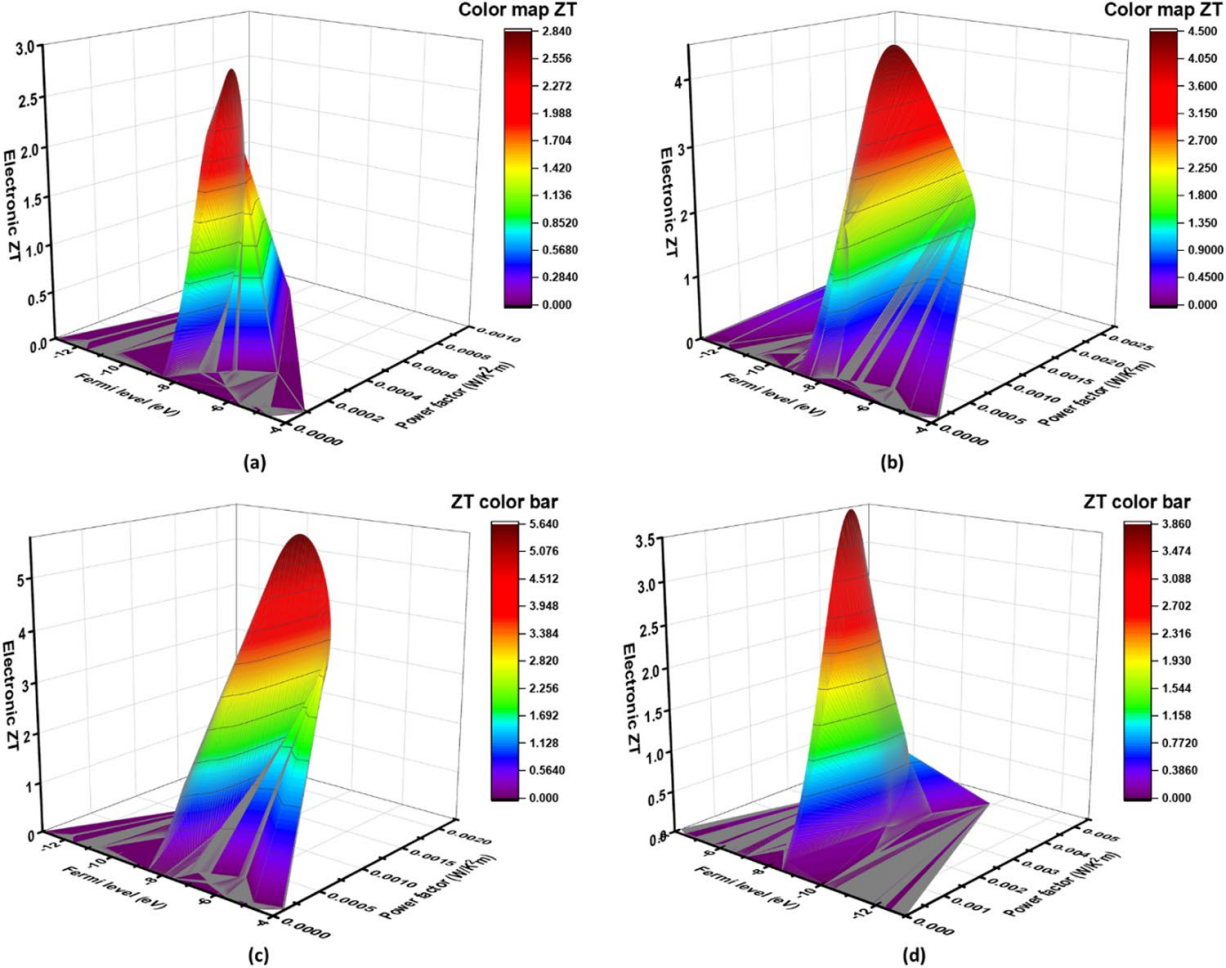
Electronic *ZT* vs power factor as a function of fermi level plots by *LanTrap*. 3D plots indicating the variation of electronic thermoelectric figure of merit *ZT* with their corresponding power factor for the band dispersions of (**a**) Cu_2_Te (**b**) Cu_1.99_Ga_0.01_Te (**c**) Cu_1.97_Ga_0.03_Te (**d**) Cu_1.95_Ga_0.05_Te. The maximum power factor of 0.0045 W m^−1^ K^−2^ was achieved in case of Cu_1.95_Ga_0.05_Te about 5.6 times the power factor of pristine Cu_2_Te (0.0008 W m^−1^ K^−2^) in the energy range −9 to −8 eV, which was attributed to the enhanced carrier conductivity due to the injection of mobile p-type carriers near the VBM.

A high-power factor is always desirable in promoting thermoelectric efficiency, since it reduces current losses and heat dissipation, thereby enhancing voltage control of the load. In order to evaluate the effect of doping on power loss, we have plotted power factor (*S*^2^*σ*) and electronic *ZT* versus fermi energy for all the compositions (Fig. 4). The maximum power factor of 0.0045 W m^−1^ K^−2^ was achieved in case of Cu_1.95_Ga_0.05_Te about 5.6 times the power factor of pristine Cu_2_Te (0.0008 W m^−1^ K^−2^) in the energy range −9 to −8 eV (please see Fig. 4a,d). Peak power factors for the compositions Cu_1.99_Ga_0.01_Te and Cu_1.97_Ga_0.03_Te were quite similar varying in the range of *S*^2^*σ* = 0.002–0.0025 W m^−1^ K^−2^ (please see Fig. 4b,d). Enhancements in the power factor as a function of Fermi energy has been plotted in Fig. 3f for all the compositions, which indicated a factor of increase of 10^2^–10^11^ near the CBM as a result of doping. Although peak power factor was the highest in case of Cu_1.95_Ga_0.05_Te, the highest electronic thermal conductivity restricted its thermoelectric efficiency (*ZT*_peak_ = 3.5), while the composition of Cu_1.97_Ga_0.03_Te exhibited highest thermoelectric performance (*ZT*_peak_ = 5.7) followed by Cu_1.99_Ga_0.01_Te (*ZT*_peak_ = 4.4) due to their low values of electronic thermal conductivities. These first-principles based thermoelectric coefficients calculations provided a valuable insight into the effect of Ga on the transport properties of Cu_2_Te.

### Morphological and phase characterization

**Figure 5:**
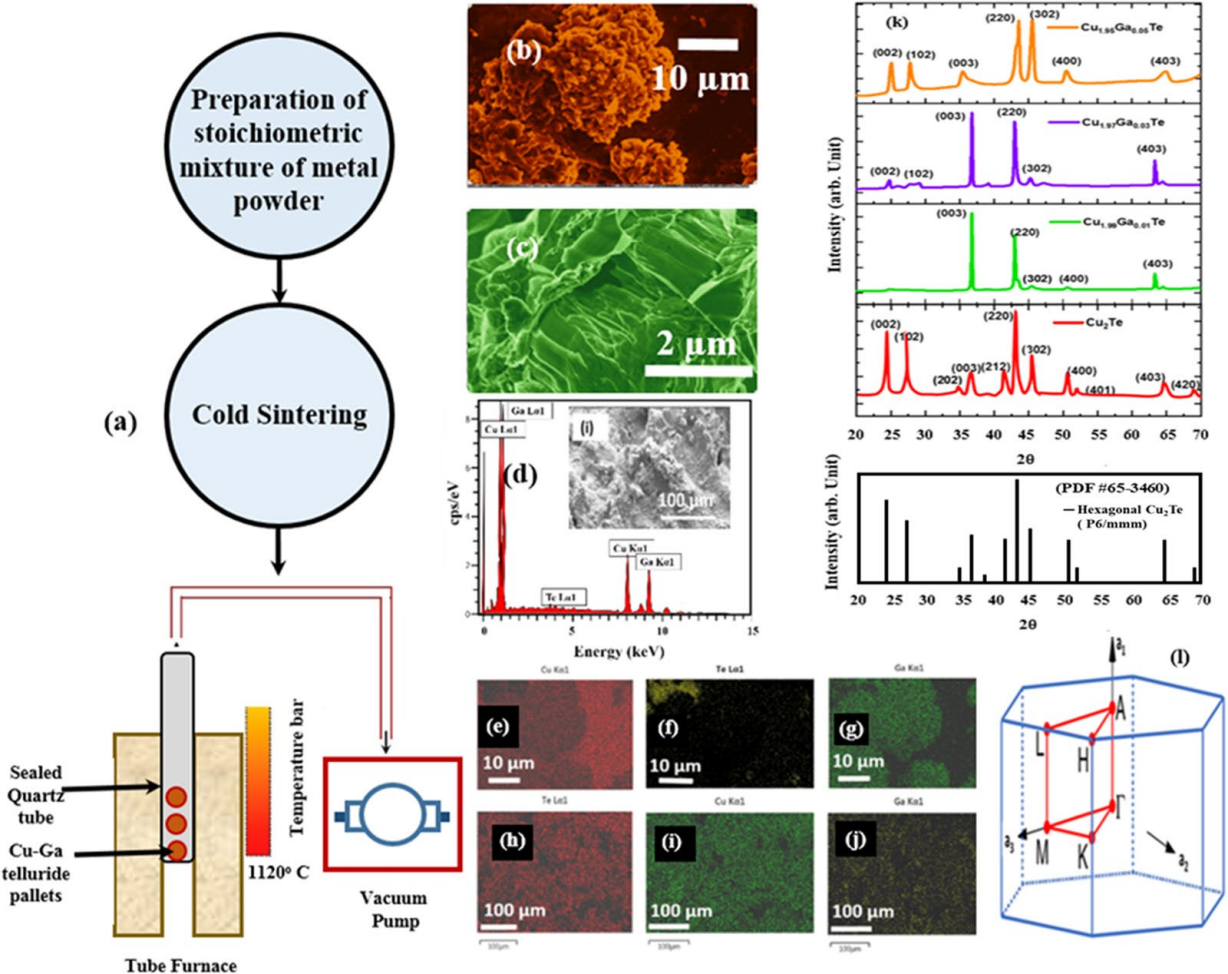
Experimental synthesis, morphological and phase examination of pristine and Ga-doped Cu_2_Te pellets. Schematic of the synthesis procedure of pellets by cold-sintering followed by direct annealing in a tube furnace under vacuum. (**b**) Representative SEM micrographs of cold-sintered Ga doped Cu_2_Te (Cu_1.97_Ga_0.03_Te in this figure) before annealing and associated EDS pattern and mapping of (**e)** K_a_ of Cu (**f**) L_a_ of Te, (**g**) K_a_ of Ga; EDS map spectrum is presented for the region shown in micrograph (**d**). The SEM micrographs showed porous irregular grains for the pellets prepared by cold sintering, the EDS maps indicated that there was not homogenous distributions of the constituent elements before annealing (**e**–**g**). (**c**) Representative SEM micrographs of the same cold sintered pellet of Ga doped Cu_2_Te (Cu_1.97_Ga_0.03_Te in this Figure) after annealing and associated EDS pattern indicating mapping of (**h**) L_a_ of Te (**i**) K_a_ of Cu (**j**) K_a_ of Ga. The SEM micrograph revealed less porosity in the irregular grains for the pellets after high temperature annealing at 1120 °C, the EDS maps indicated that there were more homogenous distributions of the constituent elements after annealing (**h**–**j**). (**k**) X-Ray diffraction patterns of the annealed pellets of Cu_2_Te, Cu_1.99_Ga_0.01_Te, Cu_1.97_Ga_0.03_Te and Cu_1.95_Ga_0.05_Te. The XRD pattern indicated resemblance to the hexagonal Cu_2_Te crystals (space group-P 6/mmm) based on PDF #65-3460 also shown in figure k. (**l**) 3D Brillouin zone for these hexagonal crystals indicating the high symmetry directions is Γ → M → K → Γ → A → L → H → A.

The densities of the samples were measured by Archimedes principle and reported in Table 2. As the Ga content increases, the densities of the samples were found to increase. The density of as sintered Cu_2_Te pellet after high temperature annealing was close to its theoretical density of hexagonal Cu_2_Te lattice^20^ (*ρ* = 7.33 *g*/*cc*). The increase in volume density due to Ga doping may be attributed to the decrease in hole concentration (shown in Table 2), since the Ga^+3^ donor gets incorporated into the intrinsic Cu vacancies in Cu_2-x_Te lattice. The morphology of sintered pellets of pristine and doped Cu_2_Te (Cu_1.97_Ga_0.03_Te in this Fig. 5) before and after annealing was characterized by using SEM. Figure 5c shows the SEM micrograph of the pellets of doped Cu_2_Te prepared by cold sintering and before high-temperature annealing. The morphology of these pellets revealed irregular grains with a porous structure (as shown in Fig. S3a–d) and the corresponding EDS maps manifested a non-uniform distribution of the elements of Cu, Te, and Ga (shown in Fig. 5e–g) as indicated by their spectral lines. After high-temperature annealing, the SEM micrographs of the pellets manifested much less porosity (please see Figs 5c and S3i–k). This may be attributed to the recrystallization process on the grains due to annealing at high temperatures, which contributed to the nucleation of defects. These defects were induced during cold-pressing (cold work) prior to annealing. High temperature annealing would allow slow diffusion of electrons from donor Ga^3+^ ion to compensate for the intrinsic Cu vacancies throughout the entire lattice of Cu_2_Te system. In addition, due to high temperature annealing, there would also be attainment of thermal equilibrium carrier concentration due to contribution of electrons from Ga resulting in the depletion of Cu vacancies. This formation of shallow donors of Ga^3+^ state due to slow annealing would result in shifting of Fermi level close to the valence band, thereby increasing the carrier density after Ga doping^41^. The EDS maps for the directly annealed pellets exhibited homogenous distribution of the constituent elements (shown in Fig. 5h–j) due to enhanced diffusion at higher temperatures. Thus, heterogeneity in the distribution of elements, which would result in thermal conductivity gradients across the sample was eliminated by annealing. The annealed pellets of all the other compositions showed similar homogenous distribution as shown in the Fig. S4, the table (l) in Fig. S4 also shows the composition of the pellets after annealing.

**Table 2:** Measured thermal conductivity for the following compounds at 300 K (κ_300K_).

	Sample	Thermal conductivity at room temperature (W/K-m) (κ_300K_)	Density (g/cc)	Hall coefficient (Ω-m/T)	Hole concentration (cm^−3^)
1	Cu_2_Te	1.05	6.93	1.87 × 10^−9^	3.33 × 10^21^
2	Cu_1.99_Ga_0.01_Te	2.81	6.94	5.99 × 10^−9^	1.04 × 10^21^
3	Cu_1.97_Ga_0.03_Te	3.13	6.98	8.18 × 10^−9^	7.64 × 10^20^
4	Cu_1.95_Ga_0.05_Te	3.58	7.01	1.10 × 10^−8^	5.65 × 10^20^

In order to study the phase purity and composition of the different phases, X-Ray diffraction patterns were also obtained for the pellets after direct annealing. Figure 5k shows the XRD pattern for pristine Cu_2_Te, Cu_1.99_Ga_0.01_Te, Cu_1.97_Ga_0.03_Te and Cu_1.95_Ga_0.05_Te. The XRD pattern of Cu_2_Te exhibited a hexagonal structure (P6/mmm) with the following lattice constants a = 0.833 nm and c = 0.722 nm (PDF #65-3460)^42^. This XRD pattern exhibited all the major peaks (002), (102), (003), (212), (220), (302), (400) and (403) of the Cu_2_Te hexagonal Newtony structure. After 1 at % doping of Ga, there was a disappearance of (002), (102) and (302), which the peak (003) exhibited the highest intensity. In Cu_2-x_Te, if Ga was incorporated in the lattice at sintering temperatures >573 K, the pellets would become more and more tellurium deficient. The XRD pattern of Cu_1.97_Ga_0.03_Te was similar to Cu_1.99_Ga_0.01_Te, this resemblance indicated that the introduction of Ga resulted in the suppression of the Te peaks of (002), (102) and (302) due to the re-evaporation of Te due to its high vapor pressure as observed previously in group I-III-VI_2_ compounds^43^. After incorporation of 5% Ga doping, there was an observation of the major peaks there was some (002), (102), (220), (302), (400) and (403). However it is interesting to note, that there was a shift noted for the peaks (002) and (302), the shifted peaks matched in 2θ values with the (112) and (312) peaks of CuGaTe_2_ (PDF# 34-1500)^44^. In addition, the peaks (002) and (102) of Te also appeared in Cu_1.95_Ga_0.05_Te, since now Te formed ordered phases with Ga and Cu which prevented evaporation of Te at high temperatures of annealing^43^. This gives us an indication of the formation some amount of intermetallic CuGaTe_2_ with the onset of 5% doping, if the doping of Ga is exceeded >5 at % then this intermetallic phase of CuGaTe_2_ would increase the thermal conductivity tremendously and hence deteriorate the thermoelectric performance. XRD pattern of the different compositions also showed asymmetry in peaks indicating the presence of microstructural defects. This may be attributed to the formation of intermetallic impurities and decrease in the Cu cation vacancy with increasing Ga content as observed from the Hall measurements.

### Thermoelectric transport property measurements

**Figure 6:**
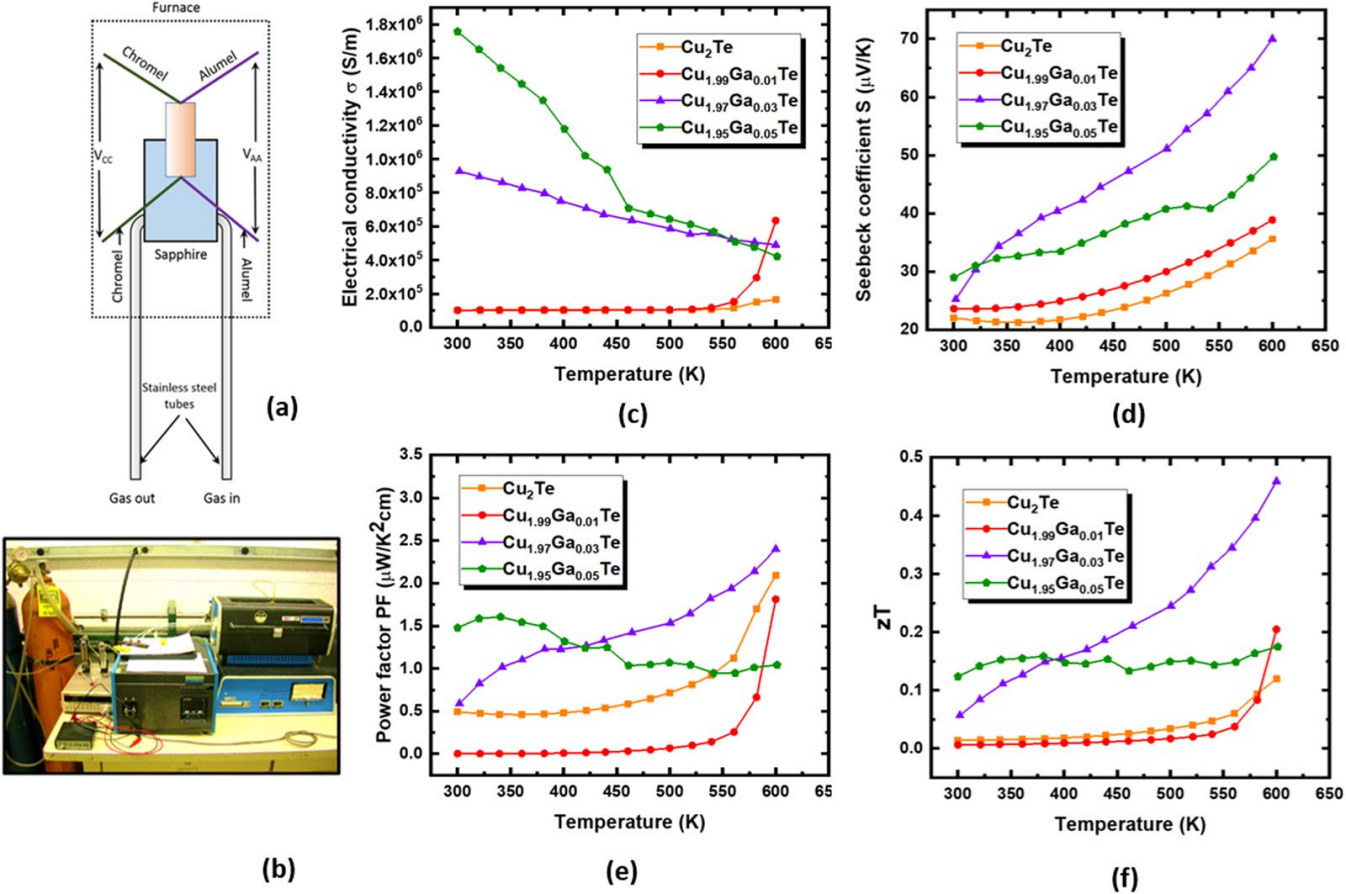
Schematic of the elevated temperature thermoelectric property measurements and thermoelectric performance of doped and pristine Cu_2_Te as a function of temperature. (**a**) An isolated flowing of gas was maintained through a thin-wall stainless steel tube bonded to the sample holder for accurate control of the probe temperature allowing a temperature gradient of ∼5 K across the sample. Reading of the temperatures was done using a four-channel National Instruments Data Acquisition System (NI-9211) thermometer. Keithley 2182 A Nanovolt meter digital Voltmeters (two) were used to measure voltage VAA (voltage across alumel legs) and VCC (voltage across chromel legs). The temperature of the sample was recorded by a type K thermocouple by connecting it to the back of a substrate which acted as a platform to support the sample. (**b**) Picture of the experimental thermoelectric property measurements setup. (**c**) Electrical conductivity (σ) (**d**) Seebeck coefficient/ thermopower (**e**) Power factor (PF) S^2^*σ* (**f**) thermoelectric figure of merit *ZT*. Ga doping increased both electrical transport (σ, S) & heat transport (κ) coefficients. However, the TE efficiency is inversely related to κ, thus Ga doping above a certain limit (~5%) degraded the thermoelectric performance. Due to the optimized combinations of a large σ (6 × 10^5^ *S*/*m*), highest S (70 μV/K) and moderate κ (3.1364 W/K-m), optimum thermoelectric performance data was observed in Cu_1.97_Ga_0.03_Te (*ZT* = 0.46 at 600 K), higher than state of the art TE materials for medium temperature applications.

**Figure 7:**
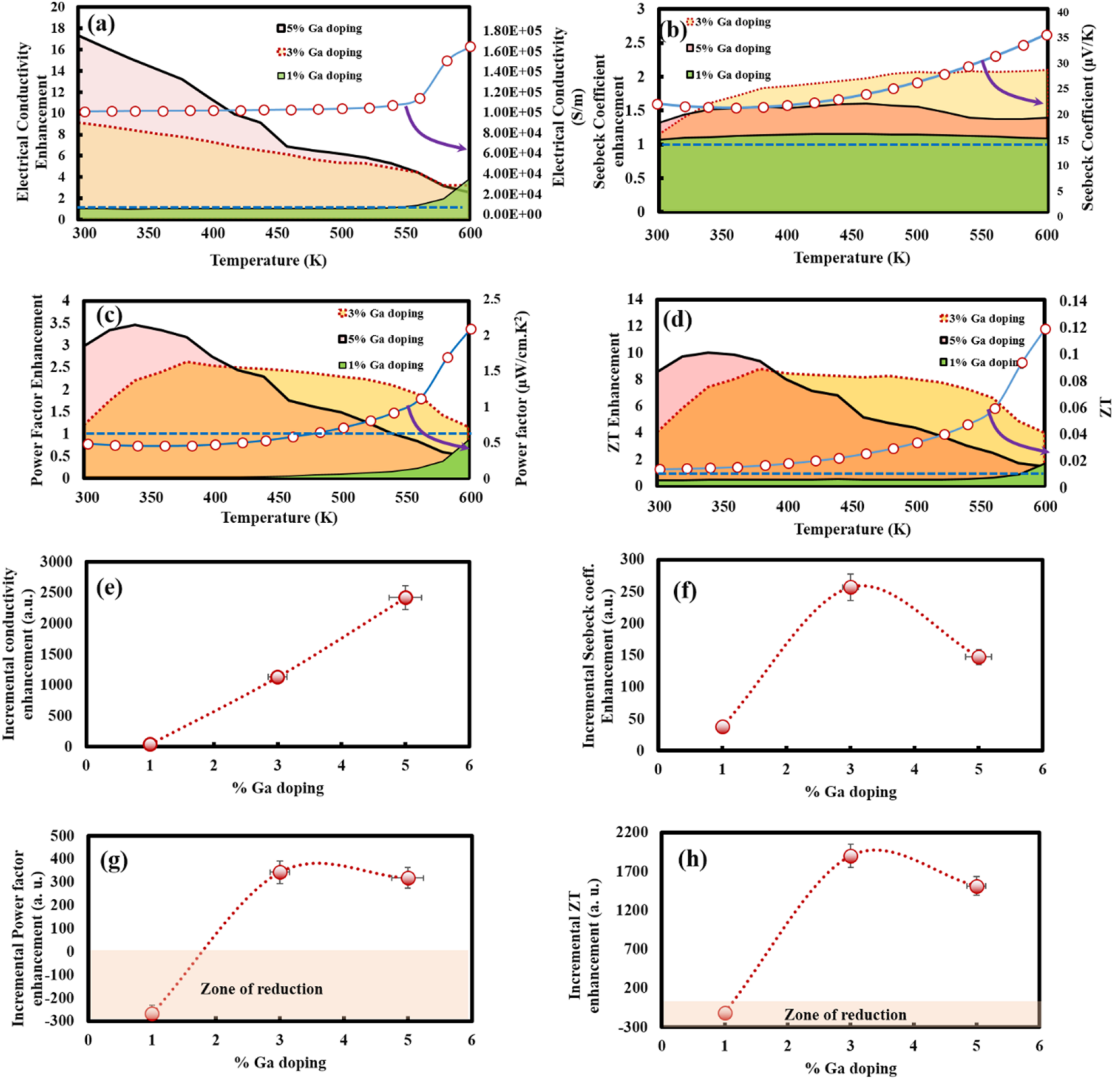
Experimental thermoelectric property measurements and enhancement in the thermoelectric performance with respect to the doping content of Ga Enhancements in transport coefficients (shown by colored area plots) with respect to temperature for doping of 1, 3 and 5% Ga. (**a**) Electrical conductivity (σ) (**d**) Seebeck coefficient/thermopower (S) (**e**) Power factor (PF) *S*^2^*σ* (**f**) thermoelectric figure of merit *ZT*. The absence of colored area regions for a particular plot (doping) essentially represents zero enhancement in that zone of temperature. Incremental Integrated enhancement over 300–600 K as function of Ga atomic % doping content for (**e**) Electrical conductivity (σ) (**f**) Seebeck coefficient/thermopower (S) (**g**) Power factor (PF) *S*^2^*σ* (**h**) thermoelectric figure of merit *ZT*. Ga doping (3 and 5%) enhanced both electrical transport (σ, S) & heat transport (κ) coefficients. In addition, better power factor was achieved after doping at medium temperatures. However, the TE efficiency is inversely related to κ, thus Ga doping above a certain limit (~5%) degraded the thermoelectric performance. Due to the optimized combinations of a large σ (6 × 10 ^5^*S*/*m*), highest S (70 μV/K) and moderate κ (3.1364 W/K-m), highest enhancement in thermoelectric performance was observed in Cu_1.97_Ga_0.03_Te (*ZT* = 0.46 at 600 K), higher than state of the art TE materials for medium temperature applications.

We examined the thermoelectric properties of all the pellets of doped and pristine Cu_2_Te after direct annealing. Figure 6(a,b) shows the schematic diagram and picture of actual experimental setup for the Seebeck coefficient measurement. Figure 6(c) shows the variation of electrical conductivity as a function of temperature in the temperature range 300–600 K. Pristine Cu_2_Te and Cu_1.99_Ga_0.01_Te exhibited a slight increase in conductivity especially after 550 K which was indicative of their semiconducting behavior. However, the compositions Cu_1.97_Ga_0.03_Te and Cu_1.95_Ga_0.05_Te revealed an enhanced electrical conductivity, which decreased with temperature thereby demonstrating their metallic range. For these two compositions, their metallic behavior exemplified a close resemblance to the narrow band IV–VI chalcogenides like CsBi_4_Te_6_^45^ and ALaCuTe_4_ (A = Na, K)^46^. This increase in electrical conductivity was attributed to the substitution of intrinsic vacancies in Cu_2-x_Te by p-type dopant Ga^3+^, found to be consistent with first principle calculations. It was also pertinent to note that the room temperature σ of pristine Cu_2_Te (σ~10^4^–10^5^ S/m) matched well with the directly annealed Cu_2_Te pellets reported earlier in literature^27^. Compared to pristine Cu_2_Te, enhancements in conductivity have been plotted as a function of temperature by colored plots for the three doping contents of 1%, 3% and 5% Ga (Fig. 7a). In case of 1% doping, there was no tangible increase in conductivity, except at temperatures over 550 K due to the enhanced mobility of the free electrons. However, in cases of 3% and 5% doping significant increases in conductivity (18-10 times maximum enhancement at 300 K) were observed due to the introduction of large number of free electrons donated by Ga^3+^. With the increase in temperature, this enhancement in conductivity decreased (Fig. 7a) due to saturation of these charge carriers, since mobility of these charge carriers decreased due to the formation of heavier bands as predicted by DFT calculations.

The Seebeck coefficients plotted in Fig. 6(d) also showed an increasing trend with temperature in the range 300–600 K for all the compositions, consistent with their p-type semiconducting behavior. For pristine Cu_2_Te, the Seebeck coefficient reached a peak value of ~45 μV/K, quite close to those reported for the directly annealed Cu_2_Te pellets at 600 K^27^. With the incorporation of Ga doping, considerable enhancement of the thermopower was further achieved with the peak S value obtained in the case of Cu_1.97_Ga_0.03_Te (S_peak_ = 70 μV/K). The peak thermopower values in Cu_1.97_Ga_0.03_Te and Cu_1.95_Ga_0.05_Te were about 2 and 1.5 times (Fig. 7b) respectively that of pristine Cu_2_Te at 600 K, which significantly promoted the thermoelectric performance. Power factor measurements, which is a yardstick for determining heat losses in TE, were also performed for the compositions as shown in Fig. 6(e). All the compositions except Cu_1.95_Ga_0.05_Te exhibited an increasing trend of power factor with temperature. This was due to the fact, that in Cu_1.97_Ga_0.03_Te, *S* exhibited a decreasing trend in the temperature range 450–550 K due to the opposing effect of minority and majority charge carriers quite common in narrow band semiconductors^26^. At room temperatures, incorporation of Ga doping of 5% enhanced the power factor to 1.5 μW/K^2^cm, which was a 25% enhancement from that obtained in the spark-plasma sintered Cu_2_Te pellets at 300 K^27^. Maximum PF of 2.3 μW/K^2^cm at 600 K was achieved in case of Cu_1.97_Ga_0.03_Te. In essence, the thermopower and the power factors was comparable to the state of the art TE materials like skutterudite CoSb_3_^47^ and CuCrSe_2_^48^. With respect to pristine Cu_2_Te, the highest enhancements (3–3.5) in PF were achieved between 300–400 K for Cu_1.97_Ga_0.03_Te, but at higher temperatures of 400–600 K, the composition Cu_1.95_Ga_0.05_Te showed the highest PF enhancements of 2–1.5 (Fig. 7c). On the other hand, 1% doping of Ga resulted in a decrease in the PF particularly in the temperature zone of 300–400 K.

For obtaining the thermal conductivities of pristine and doped Cu_2_Te, we performed PPMS thermal conductivity measurements at room temperatures. The thermoelectric figures of merit, *ZT* values for thin pellets at high temperatures are calculated using *κ* at 300 K (κ_300K_) as the thermal conductivity; owing to the fact that the thermal conductivity in copper telluride and related materials decreases with increasing temperature, hence we can use this as a conservative approximation^27,49–51^ Since this approach used room temperature thermal conductivity to predict elevated temperature *ZT*, the elevated temperature thermoelectric performance have been underestimated in this study. In addition, for Cu_2_Te related materials, the ratio of lattice thermal conductivity to total thermal conductivity ($\frac{{\kappa }_{lattice}}{{\kappa }_{total}}$) becomes close to 1 at room temperature and the contribution from electronic thermal conductivity is low. Thus total thermal conductivity (*κ*_*300K*_) for pristine and doped Cu_2_Te was used to approximate the lattice thermal conductivity. The *κ*_*300K*_ was found to increase as the content of Ga increased (shown in Table 2), with the maximum *κ*_*300K*_ of 3.58 W/K-m obtained in the case of Cu_1.95_Ga_0.05_Te. This experimental observation was again validated by our first principles calculations, which predicted an increased number of thermally active phonon modes *M* in Ga doped Cu_2_Te (disordered species like CuGaTe_2_). Since *M* is directly related to the thermal conductivity (equation 8), these disordered ionic species in Ga doped Cu_2_Te facilitated channels for heat dissipation, thus degrading the thermoelectric performance.

Finally, we presented the temperature dependent thermoelectric figures of merit, *ZT* values in Fig. 6f for all the compositions. At room temperatures, the peak *ZT* of 0.13 was observed in the case of Cu_1.95_Ga_0.05_Te, however for this composition, the *ZT* values did not increase with temperature due to its large thermal conductivity and decreased S at high temperatures. In directly annealed Cu_2_Te and Cu_1.99_Ga_0.01_Te, *ZT* values did not increase much with temperature until 500 K, when the enhanced *σ* and *S* improved its thermoelectric performance. However, due to the synergistic effects of a substantial *σ*, highest *S* and moderate *κ*_*300K*_, thermoelectric performance was found to be the best in the case of Cu_1.97_Ga_0.03_Te, again consistent with our DFT transport calculations. The *ZT* for Cu_1.97_Ga_0.03_Te increased sharply with temperature and reached its peak value of 0.46 at 600 K, higher than those reported for pseudo binary/Ag-doped/SPS/directly annealed samples (*ZT* = 0.15–0.38 at 600 K) of Cu_2_Te^21,25,27^. Moreover, the duration of annealing of 48 hours used for these pellets was much shorter than that required for synthesizing state of the art TE materials of Cu_2_Te (7 days)^27^.

The enhancement factors for the different compositions in the *ZT* values have been plotted as a function of temperature in Fig. 7d. At very low temperatures, highest *ZT* enhancement (4 to 8 times) is found for 5% doping due to the availability of a large number of charge carriers, however at temperatures of >400 K (beneficial from thermoelectric energy conversion aspect), significant enhancement in *ZT* (7 to 4 times) was achieved in the case of 3% doping due to the optimized trade-off between electronic and thermal conductivities. In addition, we also calculated the integrated incremental enhancement in transport coefficients over 300–600 K as a function of the Ga atomic % doping content in doped Cu_2_Te pellets (Fig. 7e–h) from the enhancement plots (Fig. 7a–d) by using the following methodology:

9 $${Integrated}\,{Enhancement}\,({over}\,\,300-600{K})={\int }_{{{T}}_{1}=300{K}}^{{{T}}_{2}=600\,{K}}{f}({T}){dT}-{C}$$

where, C is a constant deducted for unity enhancement (Fig. 7a–d) considered for our pristine Cu_2_Te as reference and *f*(*T*) represents the temperature dependent conductivity/Seebeck coefficient/ Power factor/*ZT* values determined experimentally. The integrated conductivity enhancement plot (Fig. 7e) indicated that it increased in direct proportion aa a function of Ga doping over the range 300–600 K, with 5% Ga doping showing the highest enhancement. Thermopower (Seebeck coefficient) enhancements when integrated over 300–600 K increased first and reached its maximum value at 3 atomic % Ga doping providing the optimum integrated thermopower enhancements (Fig. 7f). Optimum power factor and *ZT* enhancements integrated over 300K–600K was also obtained at 3 atomic % Ga doping, beyond that there was a decrease in the thermoelectric performance (Fig. 7g,h). It is interesting to note, that in case of 1% Ga doping integrated enhancement plot for power factor and *ZT* showed a negative value (reduction) as compared to pristine Cu_2_Te. This could be explained by comparing their absolute values of PF and *ZT* (Fig. 6e,f), where 1% Ga doping only improved thermoelectric performance and reduced power losses only at 600 K due to the trade-off between increased thermopower/conductivity and thermal conductivity. Thus these integrated enhancements plots revealed that significant improvements in thermoelectric performance (*ZT*, *PF and S*) at medium temperatures was possible by incorporating Ga doping but only within a certain content (3 atomic %), beyond which the increased thermoelectric power degraded thermoelectric performance and increased power losses. Hence, incorporation of Ga doping at ~3% in directly annealed Cu_2_Te provides a simple, low cost and energy-efficient (without SPS) route for the production of materials with improved thermopower, reduced power losses and enhanced TE performance.

Overall, we have presented an economical, straightforward and environment friendly route of enhancing the thermoelectric performance of Cu_2_Te through the incorporation of Ga doping (1, 3 and 5 atomic %) by direct annealing. Before experimental synthesis and characterization, first principles based DFT calculations were carried out to predict the effect of Ga doping on the bandstructure, electronic DOS and transport properties. DFT calculations predicted much steeper bands after doping along with the reduction of band gap, the electronic DOS calculations exemplified an accumulation of free electrons from the Ga^3+^ assuming a “hump” near the CBM indicative of its n-type semiconducting nature. The first principles-based transport property calculations demonstrated that Ga doping promoted *σ*, *S* which promoted thermoelectric performance. However, due to the increased number of thermally active phonon modes in disordered species (CuGaTe_2_) resulting in higher *κ*, Ga doping also exerted an adverse effect if it is not restricted within certain limits. The optimum *σ*, *S* and *κ*, as predicted by DFT calculations was achieved with 3% doping of Ga (Cu_1.97_Ga_0.03_Te), which was followed by actual experimental validation. Experimental synthesis of doped and pristine Cu_2_Te was carried out by low-cost direct annealing procedure after cold-pressing. XRD phase characterization (1, 3 and 5 atomic %) was used to identify the formation of new phases (peak shifts), particularly as the doping content of Ga increased.

The experimental thermoelectric coefficients measurements were consistent with the DFT calculations showing an increase in *σ*, *S* due to the substitution of intrinsic vacancies in Cu_2-x_Te by shallow type dopants of Ga^3+^, but thermoelectric measurements also indicated a decrease in *κ*, again similar to DFT calculations. Thus, Ga doping was found to be advantageous for enhancing the TE properties only if its content was restricted within 3 atomic %, after which there was formation of disordered species like CuGaTe_2_ (confirmed by XRD in case of 5 atomic % doping) resulting in higher thermal conductivities, thereby degrading TE performance (Fig. 1). In summary, owing to the synergistic combinations of a large *σ* (6 × 10^5^ *S*/*m*), highest *S* (70 μV/K) and moderate *κ* (3.1364 W/K-m), optimum thermoelectric performance data was observed in Cu_1.97_Ga_0.03_Te (*ZT* = 0.46 at 600 K), which is higher than the Ag-doped/ pseudo binary phase doped/ SPS prepared samples of copper telluride in this temperature range. Additionally, this synthesis methodology also involved a much shorter duration of direct annealing than in previous studies for producing high *ZT* thermoelectric materials. This study establishes Ga doped Cu_2_Te (~atomic % doping) as a promising TE material for moderate temperature ranges of 300–600 K, further scope of research lies in the transport properties study of these materials at elevated temperatures.

## Methods

### Materials

Copper powder (~200 mesh, 99.99% purity) and Gallium ingot (99.99% metals basis) were purchased from Alfa Aesar, Ward Hill, USA. Tellurium powder (~30 mesh, 99.997% purity) was purchased from Sigma Aldrich, St Louis, USA. These powders were mixed in required stoichiometric ratio and used in the synthesis of pristine and Ga-doped Cu_2_Te.

### Synthesis

Doping of Ga in Cu_2_Te was varied between the range between 1–5 at. %, since doping beyond this extent would lead to the formation of intermetallic compounds and degradation of thermoelectric properties. These powders in their elemental form were mixed thoroughly in four stoichiometric ratios (Cu:Te = 2:1, Cu:Ga:Te = 1.99:0.01:Te, Cu:Ga:Te = 1.97:0.03:Te, Cu:Ga:Te = 1.95:0.05:Te) in a crucible to form a homogenous mixture. These four mixtures of powders were pelletized by cold sintering by applying a force of 10 kg-wt on the cross-sectional area of the pellet. The diameter and the height of these cylindrical pellets after sintering were 2 cm and 5 mm, respectively. After that, the sintered pellets of Cu_2_Te, Cu_1.99_Ga_0.01_Te, Cu_1.97_Ga_0.03_Te and Cu_1.95_Ga_0.05_Te were annealed in a tube furnace at 1120 °C for 48 hours and air-cooled to room temperature slowly (Fig. 5a). The annealed pellets were then cut into square dimensions for thermoelectric property measurements.

### Density functional theory simulation

Density functional theory calculations were performed under the General Gradient Approximation (GGA) with Perdew-Burke-Ernzerhof (PBE) exchange-correlation functional, by implementation of the norm-conserving pseudopotential^52^. The plane wave pseudopotential technique (as used in the QUANTUM-ESPRESSO^53^ package within the Kohn-Sham framework using Burai 1.3 as graphical user interface) was used in our structural optimizations and electronic property calculations. During the structural optimization, a limited memory Broyden-Fletcher-Goldfarb-Shanno (LBFGS) algorithm was applied and the total energy in PBE exchange-correlation functional was used in calculating the formation energy per atom. The convergence criteria was set to 0.01 eV A^−1^ of force tolerance and 0.001 eV A^−1^ of stress tolerance.

Cu_2_Te and Ga doped Cu_2_Te (Cu_1.99_Ga_0.01_Te, Cu_1.97_Ga_0.03_Te, and Cu_1.95_Ga_0.05_Te) were modeled in a hexagonal geometry with the vacuum distance as 20 Å in a (3 × 3 × 2) supercell with a dense (5 × 5 × 5) Monkhorst-Pack k-point sampling^54^ for Brillouin-zone integration. The kinetic energy cut-off for the wave function and the charge density was set to 85 Ry and 850 Ry respectively.

The thermoelectric transport parameters were calculated from the energy band dispersion based on a solution to the Boltzmann Transport Equation (BTE) with a constant relaxation time approximation as implemented in the open source *LanTrap* code^55^. The transport coefficients like conductivity, Seebeck coefficients and thermal conductivity as a function of the Fermi energy were calculated based on the Landauer approach by evaluating of the average number of thermally active phonon modes per cross-sectional area^56^ by using a band counting algorithm. In the *LanTrap* code, the dimensions of the system used for transport coefficients calculation were L_x_ = L_y_ = 0.5 nm, L_z_ = 0.35 nm with 5 × 5 × 5 k-points along the three axes. The total number of bands were set to 198 based on the band structure calculations. The mean free path for scattering was set as 20 and 13 nm for the conduction band and valence band, respectively for pristine and doped Cu_2_Te to maintain consistency. All the transport property calculations were conducted at temperature of ~300 K.

### Morphological and phase characterization

A Rigaku –Miniflex benchtop X-ray diffractometer (Cu Kα radiation) was used to acquire the XRD pattern for identification of phases. XRD measurement scans were carried out over the 2θ range 20–70°. PDXL powder diffraction analysis software suite was used for analysis of diffraction pattern and peaks^57,58^.

The morphological characterization was performed using a Hitachi S-4800 High-Resolution Field Emission Scanning Electron Microscope. The operating parameters included an emission current of ~12 μA and accelerating voltage of ~6 kV for most of the measurements. Energy Dispersive X-ray spectroscopy (EDS) mapping was also carried out for elemental analysis of Cu, Ga, and Te, via an Oxford EDX detector unit attached to the SEM. The operating voltage was raised to ~20 kV during the EDS measurements with the emission current being ~8 μA^59,60^.

### Optical band gap and hall effect measurements

UV-Vis spectroscopy was conducted for pristine and Ga- doped pellets using a UV-3600 UV-Vis-NIR spectrophotometer (Shimadzu, Kyoto, Japan). Diffuse reflectance data was utilized for band gap measurements acquired with UV-Probe software. The wavelength was varied between 2000 nm to 200 nm with a 1 nm step size using an direct detector system with the slit width kept at 8 nm. We also estimated the room temperature hole concentration using a permanent magnet (home-made set up) of 0.31 T, for pristine and Ga-doped Cu_2_Te pellets. Hall measurements were conducted using standard four terminal methods at room temperature to compute the Hall coefficient and hole concentration.

### Elevated temperature TE measurements

High-temperature thermoelectric properties measurements were conducted using a systematic setup, where we used a horizontal tube furnace to control the temperature (see section 3.4 and associated Figures). The procedure for calculating the Seebeck coefficient was as follows: the two ends of the sample were kept fixed on the probe and extended into the tube furnace respectively. By isolated flowing of gas through a thin-wall stainless steel tube bonded to the sample holder, control of the probe temperature was achieved. This allowed a temperature gradient of ∼5 K across the sample. The thermoelectric power of the sample was measured by attaching two type K thermocouples (0.005″ in diameter) at each end of the sample. These thermocouples assisted us to measure the temperature of the sample and the voltage bias. Reading of the temperatures was done using a four-channel National Instruments Data Acquisition System (NI-9211) thermometer. Keithley 2182 A Nanovolt meter digital Voltmeters (two) were used to measure voltage VAA (voltage across alumel legs) and VCC (voltage across chromel legs).

We calculated the absolute Seebeck coefficient values based on the following method reported in literature^61^. There is a relation between the thermoelectric power of the sample, *S*_s_, with the measured sample voltages *V*_*AA*_ and *V*_*CC*_ given by:

10 $${{V}}_{{AA}}=({{S}}_{{S}}-{{S}}_{{A}})\times {\rm{\Delta }}{T}\,{\rm{and}}\,{{V}}_{{CC}}=\,({{S}}_{{S}}-\,{{S}}_{{C}})\times {\rm{\Delta }}{T}$$

where *S*_*A*_ and *S*_*C*_ represent the absolute thermoelectric power of each of the thermocouple legs, Δ*T* being the temperature difference between the two junctions. By removing Δ*T* in equation (10), we can obtain the sample’s thermoelectric power:

11 $${{S}}_{{S}}=\frac{{{V}}_{{AA}}\times {{S}}_{{C}}-{{V}}_{{CC}}\times {{S}}_{{A}}}{{{V}}_{{AA}}-{{V}}_{{CC}}}$$

The entire sample was fixed on the probe for the measurement of resistivity. A four-probe configuration was made by attaching 4 platinum wires (0.002″ in diameter) on the sample separately. The temperature of the sample was recorded by a type K thermocouple by connecting it to the back of a substrate which acted as a platform to support the sample. We used an ADCMT 6144 120 current source to supply sample current +I and −I and a Keithley 2182A digital Voltmeter read the voltages V+ and V−. During the determination of the sample resistance, we used the voltage differences due to reversed currents to factor out any induced thermal electromotive forces:

12 $${R}=\frac{{{V}}_{+}-{{V}}_{-}}{2|{I}|}$$

## Supplementary information

Supporting Information

## Data Availability

Data available upon request from Sayan Sarkar (sayan.ju92@gmail.com).
