# Supplementary material for: Synergistic effect of band convergence and carrier transport on enhancing the thermoelectric performance of Ga doped Cu2Te at medium temperatures
Source: Sci Rep. 2019 Jun 3;9:8180. doi: 10.1038/s41598-019-43911-2 (PMC6547728; doi:10.1038/s41598-019-43911-2)
Supplement: Supplementary file 1 — Supporting Information [file 41598_2019_43911_MOESM1_ESM.pdf]

Synergistic effect of band convergence and carrier transport on enhancing the thermoelectric performance of Ga doped Cu_2Te at medium temperatures

Sayan Sarkar[*1], *Prashant K Sarswat*[1], *Shrikant Saini*[2], *Paolo Mele*[3], *Michael L Free*[1]

[1]*Department of Metallurgical Engineering, University of Utah, Salt Lake City, Utah, United States*

[2]*Department of Mechanical and Control Engineering, Kyushu Institute of Technology, Kitakyushu , Japan*

[3]*Shibaura Institute of Technology, SIT Research Laboratories, Toyosu, Koto-Ku, Tokyo, Japan.*

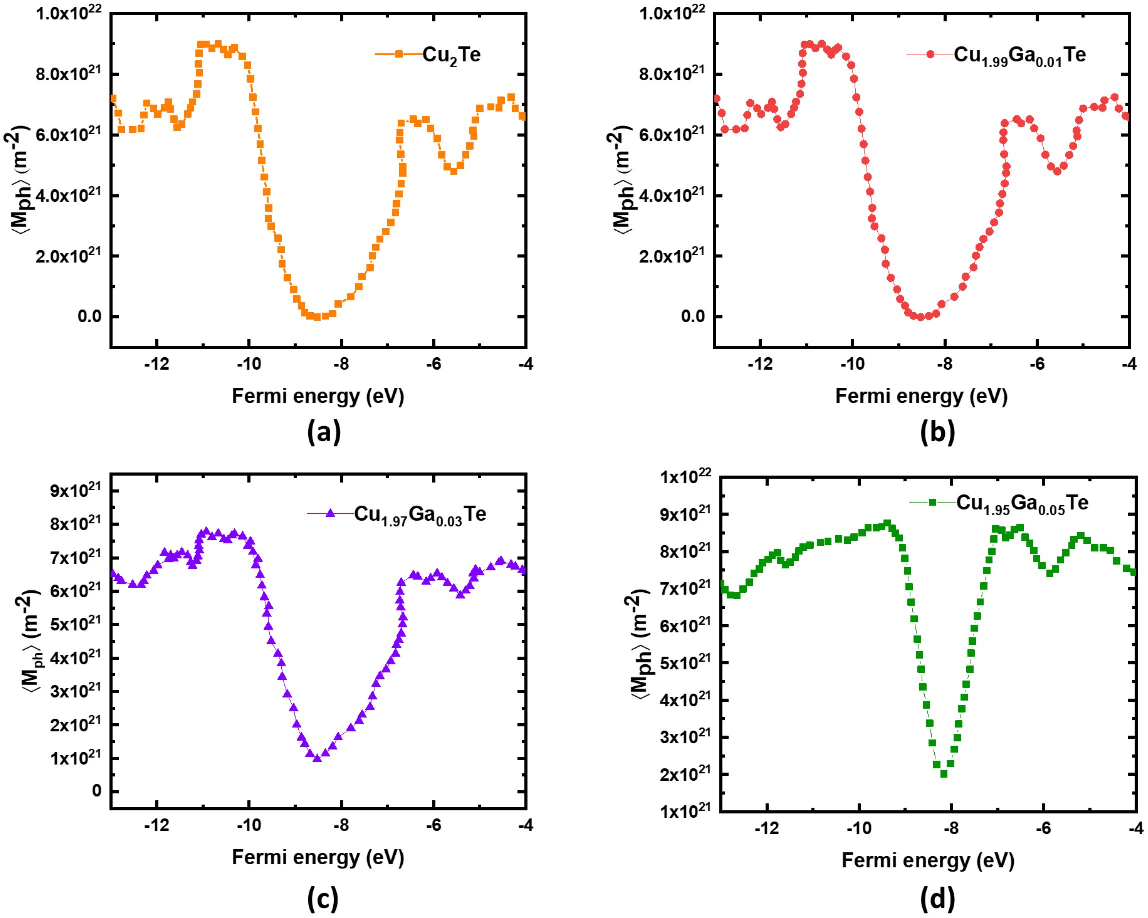

Figure S1: **First principle calculations of distribution of modes for thermoelectric transport from the band dispersions of pristine and Ga-doped Cu_2Te.**

Average number of conducting phonon modes per cross-sectional energy ($\langle M_{ph} \rangle$)versus vs fermi level for (a) Cu_2Te (b) $Cu_{1.99}Ga_{0.01}Te$ (c) $Cu_{1.97}Ga_{0.03}Te$ (d) $Cu_{1.95}Ga_{0.05}Te$. These distributions of modes were calculated from the bandstructures of the corresponding crystals using the thermoelectric transport calculator tool *LanTrap*.

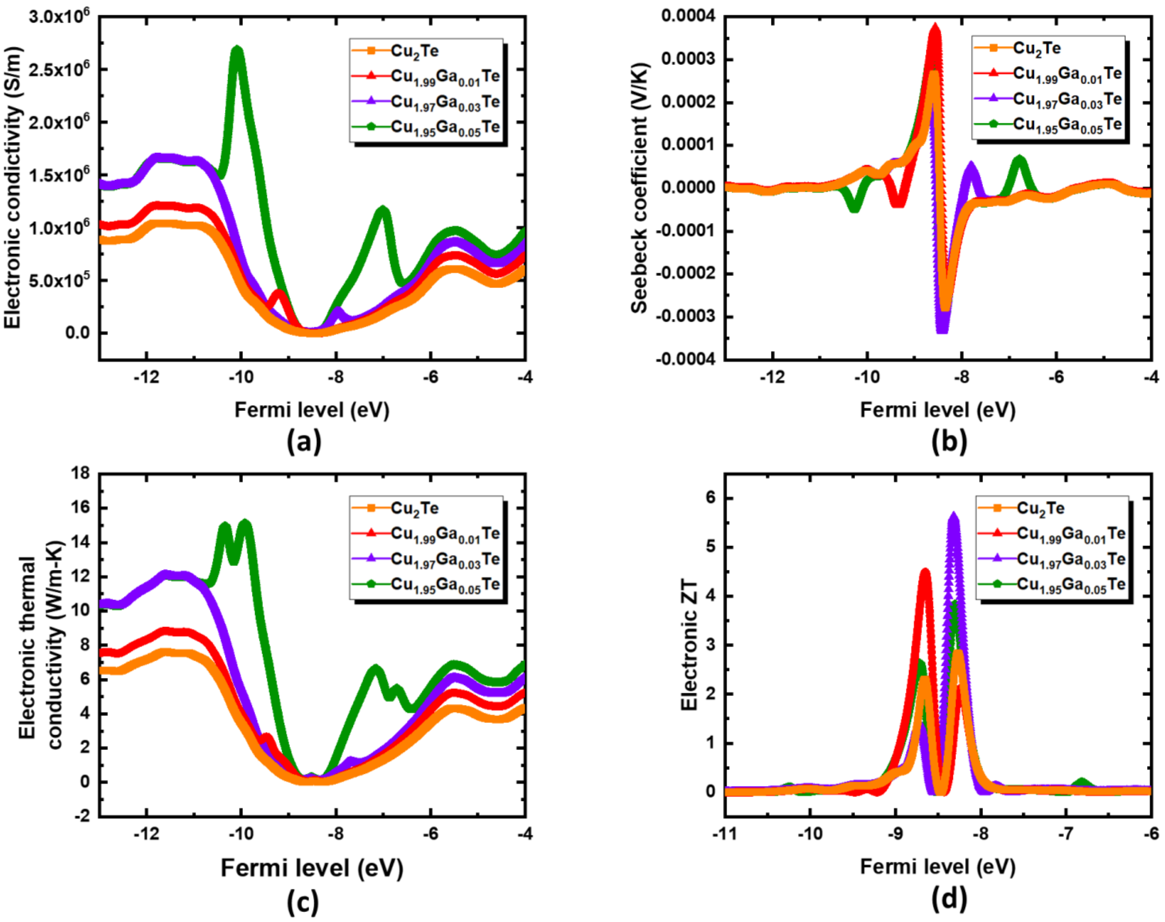

Figure S2: **First principle calculations of thermoelectric transport properties by *LanTrap.***

(a) Variation of electrical conductivities as a function of fermi level (b) Variation of Seebeck coefficients as a function of fermi level (c) Variation of electronic thermal conductivity as a function of fermi level (d) Variation of electronic ZT as a function of fermi level for Cu_2Te, $Cu_{1.99}Ga_{0.01}Te$, $Cu_{1.97}Ga_{0.03}Te$ and $Cu_{1.95}Ga_{0.05}Te$.

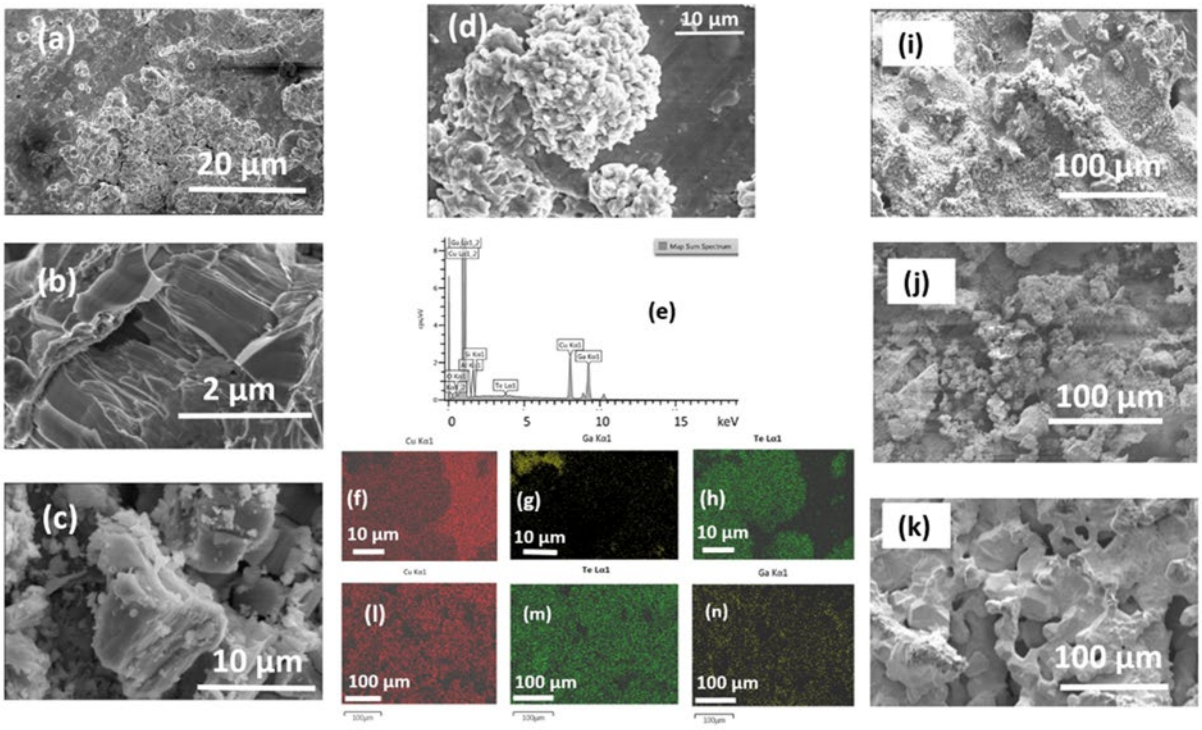

Figure S3: **Morphological examination of Ga-doped Cu_2Te pellets before and after annealing**

(a-b-c-d) Representative SEM micrographs of Ga doped Cu_2Te ($Cu_{1.97}Ga_{0.03}Te$ in this figure) at different resolutions before annealing and associated EDS pattern (e) and mapping of (f) K_a of Cu (g) K_a of Ga, (h) L_a of Te; EDS map spectrum is presented for the region shown in micrograph (d). The SEM micrographs showed porous irregular grains for the pellets prepared by cold sintering, the EDS maps indicated that there was not homogenous distributions of the constituent elements before annealing. (i-j-k) Representative SEM micrographs of the same cold sintered pellet of Ga doped Cu_2Te ($Cu_{1.97}Ga_{0.03}Te$ in this Figure) after annealing at different locations and associated EDS pattern indicating mapping of (l) K_a of Cu (m) L_a of Te, (n) K_a of Ga. The SEM micrograph revealed less porosity in the irregular grains for the pellets after high temperature annealing, the EDS maps indicated that there was more homogenous distributions of the constituent elements after annealing.

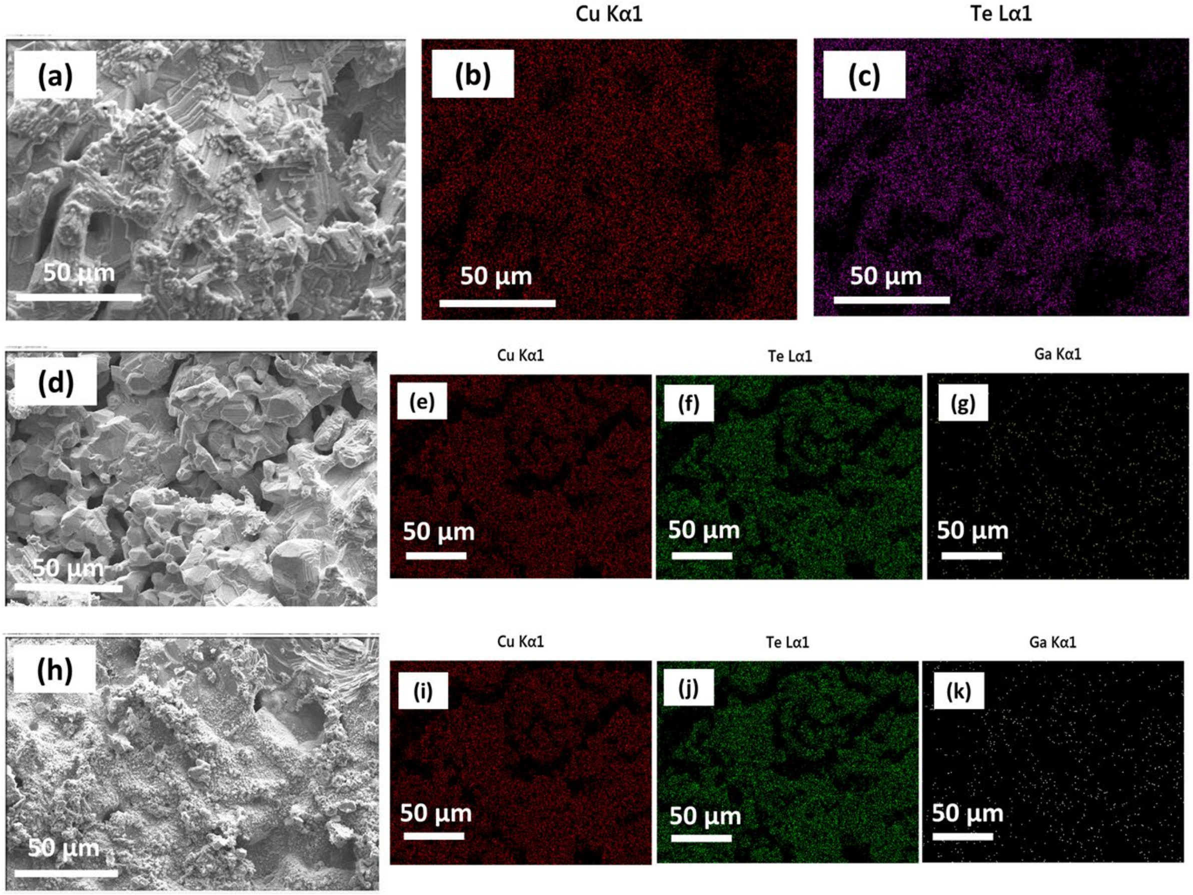

Sample	Atomic %		
	Cu	Ga	Te
Cu_2Te	61.7	-	38.3
$Cu_{1.99}Ga_{0.01}Te$	60.3	0.98	38.72
$Cu_{1.97}Ga_{0.03}Te$	56.4	2.6	41
$Cu_{1.95}Ga_{0.05}Te$	54.8	4.7	40.5

(l)

Figure S4: **Morphological examination of pristine & Ga-doped Cu_2Te pellets after annealing**

SEM micrographs of (a) pristine Cu_2Te, associated EDS maps of (b) K_a of Cu (c) L_a of Te; SEM micrographs of (d) $Cu_{1.99}Ga_{0.01}Te$, associated EDS maps of (e) K_a of Cu (f) L_a of Te (g) K_a of Ga; SEM micrographs of (h) $Cu_{1.95}Ga_{0.05}Te$, associated EDS maps of (i) K_a of Cu (j) L_a of Te (k) K_a of Ga (l) composition of samples as detected by EDS.

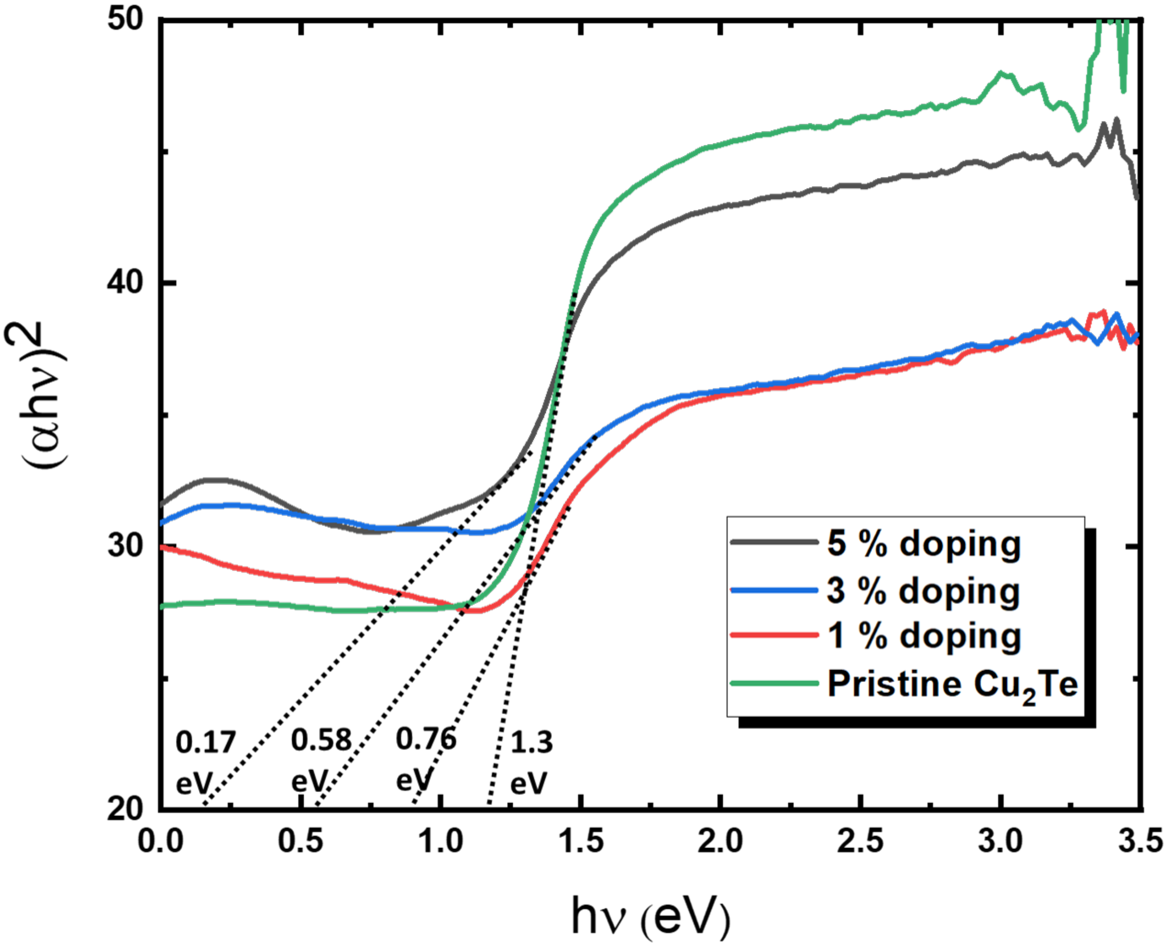

Figure S5: **Optical Band Gap measurements of pristine & Ga-doped Cu_2Te pellets after annealing**

Tauc plots for Cu_2Te, $Cu_{1.99}Ga_{0.01}Te$, $Cu_{1.97}Ga_{0.03}Te$ and $Cu_{1.95}Ga_{0.05}Te$ after annealing. The extrapolated band gaps (determined by tangents from the linear portion) indicate that optical band gap decreases as Ga content is increased.
